# Loss of cyclase-associated protein 2 alters actin cytoskeleton organization and biomechanical properties of the ocular lens

**DOI:** 10.1242/jcs.264491

**Published:** 2026-05-26

**Authors:** Sepideh Cheheltani, Megan Coffin, Velia M. Fowler

**Affiliations:** Department of Biological Sciences, University of Delaware, Newark, DE 19716, USA

**Keywords:** Cyclase-associated protein 2 CAP2, Actin-cytoskeleton, Lens fibers, Lens biomechanics, Tropomodulin, Plastin

## Abstract

Cyclase-associated protein 2 (CAP2) is a conserved actin-binding protein that promotes actin filament (F-actin) turnover by disassembling ADF/cofilin-decorated filaments, supporting F-actin remodeling in differentiating cells and tissues. In the ocular lens, the actin cytoskeleton is crucial for maintaining tissue biomechanical properties during fiber cell maturation. To assess the role of CAP2 in the lens, we examined lens-specific CAP2 knockout (*CAP2^cKO^*) mice at cellular and tissue levels. *CAP2^cKO^* lenses were normal in size, shape and transparency but exhibited increased stiffness under compression and enhanced recovery after load removal. Although total actin and F-actin levels were unchanged, immunofluorescence revealed higher levels for tropomodulin 1 (Tmod1, F-actin pointed-end capping), tropomyosin isoform 3.5 (Tpm3.5, F-actin stabilizing) and T-plastin (also known as PLS3; F-actin bundling) in F-actin-rich membrane protrusions of mature fibers, whereas that for α-actinin-1 (F-actin cross-linking) was reduced. These findings suggest that CAP2 loss disrupts F-actin remodeling, indirectly promoting filament stabilization through Tpm3.5 binding, Tmod1 capping and T-plastin bundling. Consequently, F-actin networks become stiffer and more resilient, altering lens biomechanics. This study provides the first evidence that CAP2 maintains cell biomechanical properties in a non-muscle tissue through influencing the composition of actin cytoskeleton networks

## Teaser

**Summary:** CAP2 loss alters the actin cytoskeleton through increased F-actin stabilization by Tmod1 and Tpm3.5, and filament bundling by T-plastin, leading to stiffer lenses.

## INTRODUCTION

Cyclase-associated protein 2 (CAP2) is a conserved actin-regulatory protein (Peche et al., 2007; Rust et al., 2020) that modulates actin filament (F-actin) turnover by enhancing the depolymerization and severing of ADF/cofilin-bound F-actin from the pointed ends (Kotila et al., 2019; Ono, 2013; Shekhar et al., 2019). Beyond facilitating cofilin-mediated actin turnover, CAPs are recognized as a multifunctional regulator of actin dynamics. They catalyze nucleotide exchange on G-actin to promote actin monomer recycling (Kotila et al., 2018), interact with actin nucleators, such as formins, and Ena/VASP, and coordinate actin filament organization through interactions with signaling pathways (Kepser et al., 2021). These diverse roles highlight CAPs as central modulators of actin homeostasis and cytoskeletal remodeling. CAP2 has been shown to play tissue-specific roles in organizing cytoskeletal architecture and supporting cellular functions (Field et al., 2015; Kosmas et al., 2015; Ono, 2013; Rust et al., 2020). In striated muscle, CAP2 localizes near the pointed ends of the thin filaments and is essential for proper myofibril assembly (Kepser et al., 2019); its deletion leads to disorganized actin networks, dilated cardiomyopathy and impaired contractility (Colpan et al., 2021; Field et al., 2015; Peche et al., 2013). In non-muscle tissues, such as the epidermis, CAP2 regulates cortical actin at cell junctions and promotes cell migration during wound healing (Kosmas et al., 2015; Peche et al., 2007). In neurons, CAP2 is a key regulator of actin dynamics, controlling dendritic spine morphology and synaptic plasticity by binding cofilin proteins and inhibiting the actin nucleator INF2, mechanisms essential for proper spine maturation and cognitive function (Heinze et al., 2022; Kumar et al., 2016; Pelucchi et al., 2020). However, whether CAP2 supports cytoskeletal and mechanical functions in other specialized, non-contractile cells and tissues remains unclear. Here, we examine how CAP2 contributes to F-actin organization and biomechanics in the ocular lens, a transparent, deformable tissue that depends on a specialized F-actin network for cell differentiation and maturation.

The lens is a transparent spherical tissue in the anterior chamber of the eye, located behind the iris and in front of the vitreous humor (Lovicu and Robinson, 2004). It is composed of two types of cells, a monolayer of cuboidal epithelial cells on the anterior surface that overlays concentric layers of long, thin fiber cells extending from the anterior to the posterior poles, forming the vast majority of the lens mass (Bassnett and Mataic, 1997). The entire lens is encapsulated by a thin collagenous basement membrane, known as the capsule, which is produced by lens epithelial cells (Bassnett et al., 1999; Danysh and Duncan, 2009; Haddad and Bennett, 1988) (Fig. S1A). Lens function is dependent on two properties, transparency and biomechanics. The biomechanical properties of the lens, particularly its stiffness and elasticity, are essential for maintaining optical clarity and enabling accommodation (changes in lens shape to enable near and far focusing) (Cheng et al., 2019; Gupta et al., 2023). Age-related changes in lens stiffness contribute to the development of presbyopia in primates (loss of lens ability to accommodate), underscoring the importance of understanding the molecular mechanisms that regulate lens mechanics.

The eye lens presents a distinctive cellular context in that it continues to grow throughout life by the addition of new fiber cells at the equator (Fig. S1A) (Bassnett and Šikić, 2017). This process is mediated by the proliferation of equatorial lens-epithelial cells, followed by their differentiation and elongation into secondary fiber cells, which are then deposited in concentric shells around older primary cells that form the core of the lens (lens nucleus) (Fig. S1A) (Bassnett and Šikić, 2017). Changes in the rate of epithelial proliferation or differentiation can alter the number of fiber cells added, thereby influencing final lens size (Šikić et al., 2017). Thus, disruptions in genes crucial for lens development or fiber cell organization often lead to smaller lenses in adult mice, demonstrating how early defects can have lasting effects on lens size (Graw, 2019; Li et al., 2024).

Another unusual aspect of the lens is that lens fiber cells are non-contractile and non-motile, yet they rely on dramatic actin filament rearrangements during differentiation and maturation throughout life to maintain lens transparency. During maturation, lens fiber cells develop specialized paddles and interlocking membrane interdigitations enriched in F-actin networks with diverse associated actin-binding proteins (ABPs) (Fig. S1B) (Bassnett and Šikić, 2017; Cheng et al., 2016b; Šikić et al., 2015). This complex architecture is organized by a host of ABPs that regulate filament assembly, turnover and organization (Cheng et al., 2017). Previous research investigating the roles of ABPs in lens actin networks and biomechanical properties has revealed that the reduction or deletion of the F-actin-stabilizing proteins, tropomyosin isoform 3.5 (Tpm3.5, encoded by *Tpm3*) and tropomodulin 1 (Tmod1), leads to F-actin network rearrangements and altered lens biomechanical properties with reduced lens stiffness (Cheng et al., 2018, 2016b; Fischer et al., 2000; Gokhin et al., 2012). Tpms and Tmods are actin-stabilizing proteins that protect F-actin from ADF/cofilin-mediated disassembly and depolymerization in a variety of cells (Gunning et al., 2015; Jansen and Goode, 2019; Ono and Ono, 2002). However, the role of actin disassembly proteins, such as ADF/cofilin and CAPs, in the turnover and rearrangement of the lens actin cytoskeleton network is unknown (Bamburg and Bernstein, 2010; Cheng et al., 2017). A key question and the central focus of this study is what role CAP2 plays in regulating actin networks in the lens and how it interfaces with other ABPs to maintain the unique architecture of fiber cells.

Notably, CAP2-knockout (KO) mice develop microphthalmia (small eye) (Field et al., 2015), indicating that CAP2 might be important for normal lens growth or architecture. Here, we have investigated how CAP2 affects lens size, transparency and biomechanics, and how CAP2 fits into the network of ABPs in the lens. We ask whether CAP2 mediates actin filament remodeling in fiber cells and cooperates or antagonizes with known lens ABPs to assemble and maintain the cytoskeletal F-actin networks. By investigating the role of CAP2 within the broader ecosystem of filament regulators, we aim to understand how the actin cytoskeleton of the lens is affected in the absence of a key actin regulatory protein and provide insight into the general principles by which tissue-specific actin networks are organized to support specialized cellular functions. In the following, we present a focused analysis of the role of CAP2 in the lens, detailing its expression and functional impact on fiber cell organization and lens biomechanics, thereby shedding light on how this actin regulator contributes to the complex requirements of lens flexibility.

## RESULTS

### CAP2 protein is absent in *CAP2^cKO^* mice lens

**Fig. 1.**
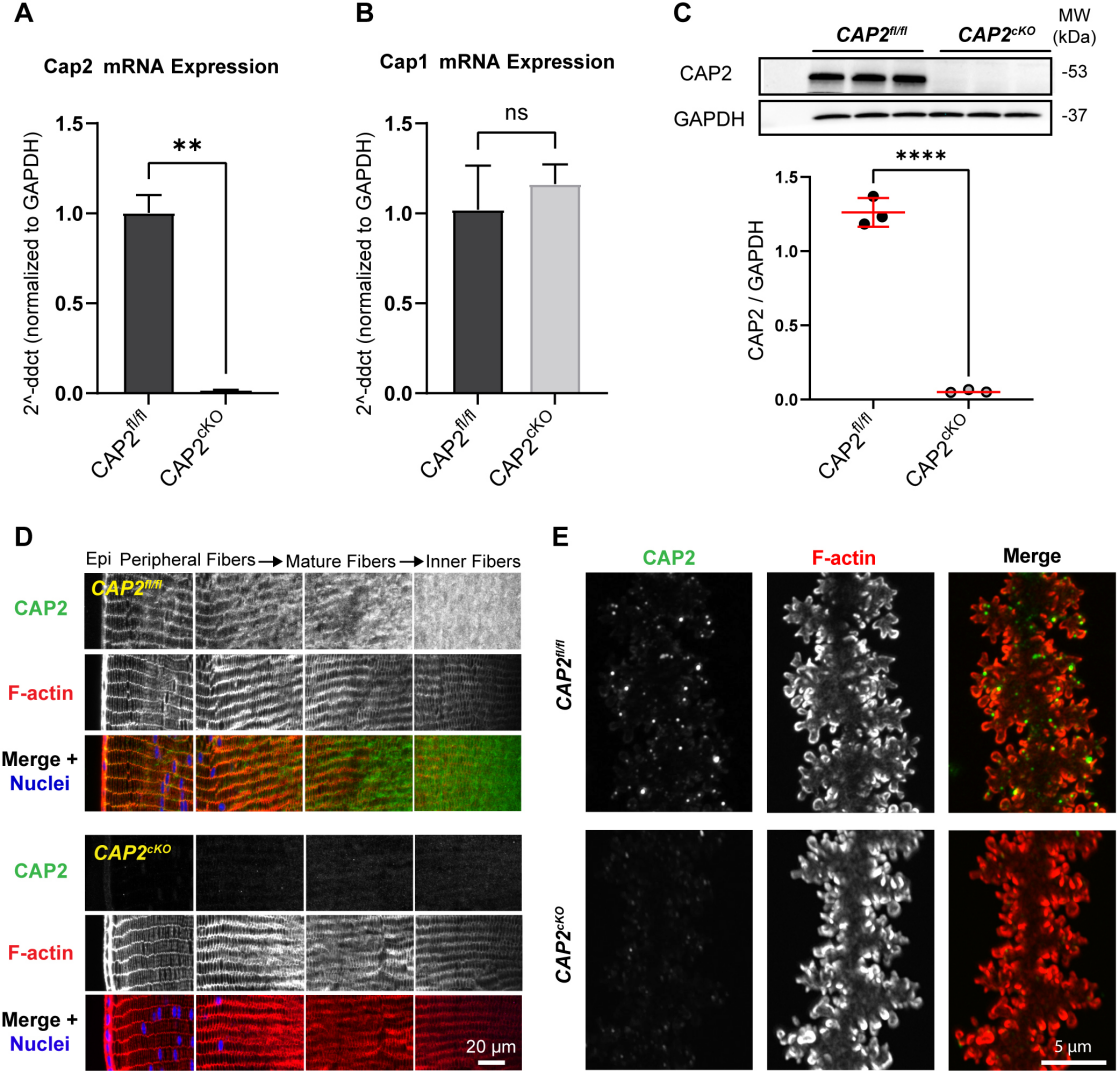
**CAP2 is not present in *CAP2^cKO^* mouse lenses.** (A) RT-qPCR from three *CAP2^cKO^* and *CAP2^fl/fl^* mouse lenses reveals a significant decrease in transcript levels in KO samples. (B) RT-qPCR from three *CAP2^cKO^* and *CAP2^fl/fl^* mouse lenses reveals no significant changes in mRNA levels of Cap1. GADPH was used as a housekeeping gene. (C) Western blots of *CAP2^cKO^* and *CAP2^fl/fl^* lenses reveal the absence of CAP2 protein in KO samples. GAPDH is used to normalize protein levels. Plots reflect mean±s.d. of *n*=3 independent samples per genotype. **P<0.01; ****P<0.0001; ns, not significant (two-tailed unpaired *t*-test). (D) Immunostaining of equatorial cryosections of 8-week-old *CAP2^fl/fl^* and *CAP2^cKO^* lenses stained with anti-CAP2 rabbit polyclonal antibody (green), rhodamine phalloidin for F-actin (red), and Hoechst for nuclei (blue). In control lens fiber cells, CAP2 staining appears enriched with F-actin on the broad sides, at the vertices, and diffuse in the cytoplasm. (E) Immunostaining of single fiber cells from 8-week-old *CAP2^cKO^* and *CAP2^fl/fl^* lenses for CAP2 (green) and F-actin (red). Images are maximum intensity projections and representative of *n*=3 mice per genotype. Scale bars: 20 μm (D), 5 μm (E).

The survival rate for CAP2 global KO mice is very low, with the few that survive after birth exhibiting multiple defects, including severe cardiomyopathy, and typically dying before 10 weeks of age (Field et al., 2015). Therefore, to investigate the function of CAP2 in the adult mouse lens and to circumvent potential systemic effects of global CAP2 deletion, we utilized a conditional knockout strategy to delete CAP2 in lens epithelium and fiber cells using the Cre-Lox system (Fig. S2). Specifically, we used the *MLR10-Cre* transgenic mouse, which induces CRE expression in lens epithelial and fiber cells at embryonic day 10.5 (E10.5) (Zhao et al., 2004), to generate a lens-specific CAP2 KO mouse (Lam et al., 2019). RT-qPCR and western blot analysis confirmed the absence of *Cap2* mRNA and protein in *CAP2^fl/fl Tg (Cryaa-Cre)10Mlr^* (hereafter referred to as *CAP2^cKO^*) lens samples (Fig. 1A,C). To confirm that the deletion of CAP2 is lens specific, we immunostained 8-week-old lens frozen sections in the equatorial and sagittal orientations with anti-CAP2 rabbit antibody. Immunostaining of mouse eye sections revealed that CAP2 is expressed in the lens, cornea and retina (Fig. S3A). Low-magnification confocal images demonstrated that the CAP2 signal was absent in *CAP2^cKO^* lens, in contrast to the robust CAP2 staining observed in non-lens tissues, such as the ciliary body (Fig. S3B). Additionally, although we focused on CAP2, mice express both *Cap1* and *Cap2* genes (Rust et al., 2020). To assess potential compensatory mechanisms, we examined whether *Cap1* expression is altered in *CAP2^cKO^* lenses. However, we detected no change in *Cap1* mRNA levels in the absence of *Cap2* (Fig. 1B). According to the iSyTE database of lens gene expression (Lachke et al., 2012), *Cap2* is more abundantly expressed and highly enriched in the lens compared to *Cap1*. Thus, it is unlikely that *Cap1* expression compensates for the loss of CAP2 in KO lenses.

### CAP2 is present in puncta near F-actin-rich protrusions of lens fiber cells

To study the role of CAP2 in the lens actin cytoskeleton, we first examined the expression and localization of CAP2 protein in mouse lens cryosections (Fig. 1D). In equatorial cryosections, CAP2 labeling was prominent in lens fiber cells with minimal signal in lens epithelial cells. In fiber cells, CAP2 staining appeared enriched in the same location as F-actin on the broad sides and at the vertices, as well as being diffuse in the cytoplasm. Additionally, CAP2 was localized with F-actin near the membrane in both differentiating and maturing fiber cells, but was more abundant in the cytoplasm of inner cortical fiber cells (Fig. 1D). Taking a closer look at the subcellular localization of CAP2 in mature fiber cells revealed that CAP2 appeared as puncta near the F-actin-rich protrusions (Fig. 1E). To quantify the spatial association between CAP2 puncta and F-actin observed in Fig. 1E, we determined the extent of colocalization using Manders' coefficient. The Manders' coefficient for CAP2 relative to F-actin was 0.613±0.139 (mean±s.d.), indicating that∼60% of CAP2 signal overlaps F-actin-positive regions, consistent with partial spatial association of CAP2 puncta with actin filaments. Together, these data indicate that CAP2 is enriched in the cytoplasm of lens inner fiber cells, with a subset of CAP2 puncta positioned near F-actin–rich membrane protrusions in mature cortical fibers, consistent with partial spatial association with the actin cytoskeleton.

### *CAP2^cKO^* lenses are normal in size and shape but exhibit increased stiffness

**Fig. 2.**
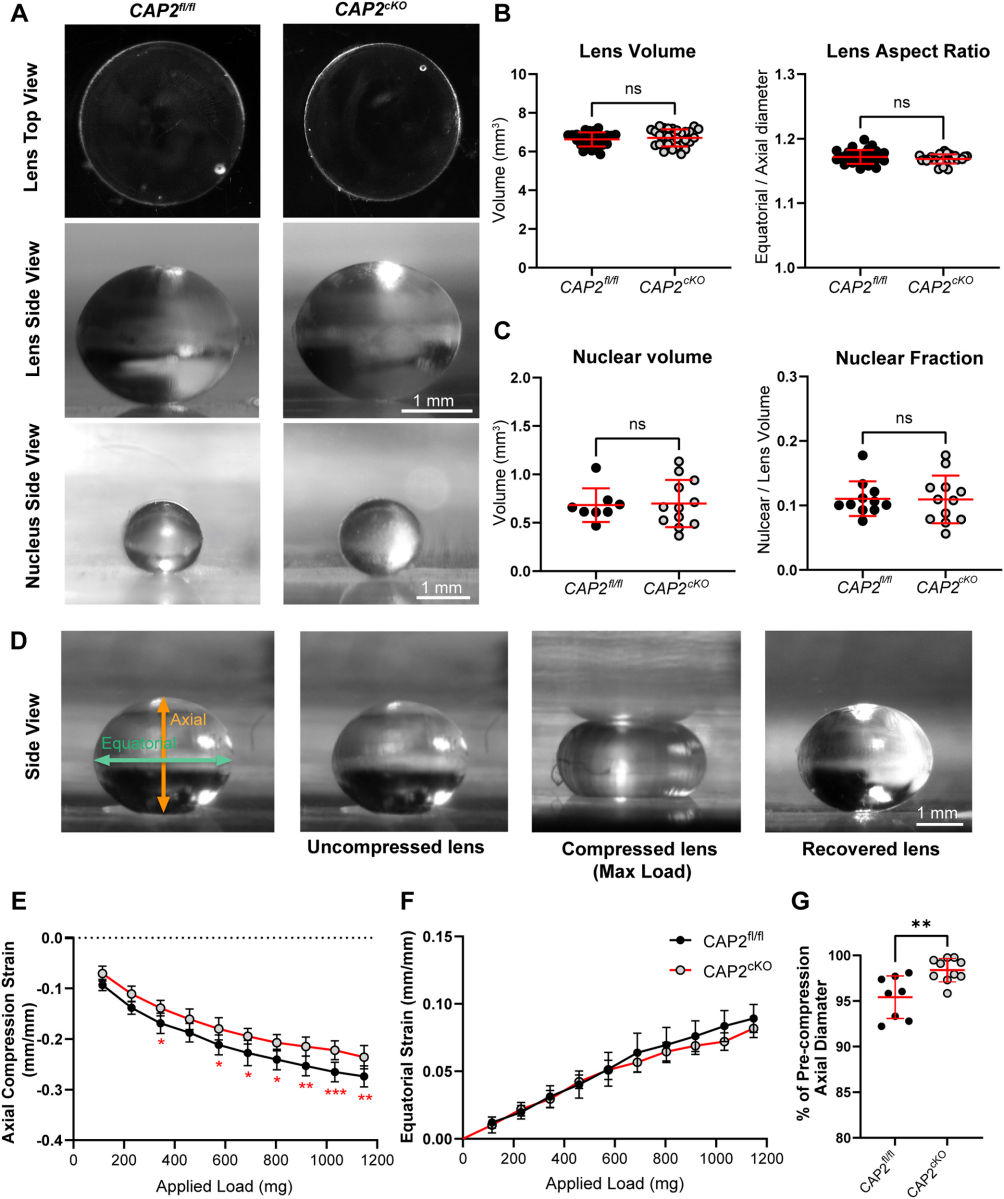
***CAP2^cKO^* lenses have normal morphology but are stiffer and more resilient.** (A) Images of freshly dissected 8-week-old *CAP2^cKO^* and *CAP2^fl/fl^* lenses. All lenses were transparent without obvious opacities. (B) Whole lens volume and aspect ratio (equatorial to axial diameter ratio) from 8-week-old control and mutant mice show no significant differences in whole lens size and shape. (C) Nuclear fraction and volume show no significant difference in mutant lenses. Plots show mean±s.d. of 12–15 lenses from at least seven biological replicates per genotype. (D) Side view images of uncompressed, compressed, and recovered lenses. (E,F) Plots of axial and equatorial strain (d−d0)/d0 show increased axial strain in *CAP2^cKO^* lenses, especially at high loads. (G) *CAP2^cKO^* lenses had increased resilience, calculated as ratio of the pre-compression to post-compression axial diameter. Plots reflect mean±s.d. of eight lenses from at least five mice per genotype. Scale bars: 1 mm. *P<0.05; **P<0.01; ***P<0.001; ns, not significant (two-way ANOVA with Sidak's multiple comparisons test).

To evaluate lens morphology in *CAP2^cKO^* mice, we conducted a comprehensive examination of whole lens morphology by analyzing freshly dissected lenses from 8-week-old *CAP2^cKO^* and control mice (*CAP2^fl/fl^*). Our initial image analysis revealed that the lenses from *CAP2^cKO^* mice were transparent, with no apparent opacities (Fig. 2A). Morphometric analyses further revealed no significant alterations in either lens total volume or shape (aspect ratio) in *CAP2^cKO^* lenses compared to control littermates for either male or female mice (Fig. 2A,B). Additionally, the nuclear volume and nuclear fraction remained unchanged in the mutant lenses (Fig. 2A,C). Taken together, these findings argue against a gross defect in overall lens growth or development due to CAP2 loss-of-function in the lens, suggesting that CAP2 is unlikely to play a primary role in determining lens size or overall morphogenesis.

To evaluate whether the absence of CAP2 alters the biomechanical properties of the lens, we conducted a coverslip compression assay to measure lens stiffness and ability to recover (Fig. 2D) (Cheng et al., 2016a). Our analysis revealed a significant decrease in axial compressive strain in *CAP2^cKO^* lenses, particularly at higher loads, indicating that *CAP2^cKO^* lenses are stiffer than control lenses under compression (Fig. 2E,F). Additionally, we evaluated lens shape recovery after load removal by calculating the ratio of the pre- to post-loading axial diameter, referred to as resilience. Our findings demonstrated a significant improvement in recovery in *CAP2^cKO^* lenses, with a mean recovery to 98.39±1.28% (mean±s.d.) of the pre-loading axial diameter, as compared to 95.42±2.337% for the control lenses (Fig. 2G). These results are unexpected, as previous work on mice with reduced levels of ABPs, such as Tpm3.5, Tmod1 or the lens intermediate filament protein CP49 (also known as BFSP2), led to softer lenses with decreased resilience, notably in Tpm3.5-deficient lenses (Cheng et al., 2018; Gokhin et al., 2012).

Studies of decapsulated lenses have demonstrated that the capsule contributes to lens shape retention and resilience (Mekonnen et al., 2023; Wilde et al., 2012). To assess whether changes in capsule thickness might contribute to the increased stiffness and enhanced recovery of *CAP2^cKO^* lenses, we measured anterior capsule thickness in both *CAP2^cKO^* and *CAP2^fl/fl^* 8-week-old mouse lenses (Fig. S4A–C). Image analysis revealed no significant differences between genotypes (Fig. S4C), indicating that capsule thickness is unaffected by CAP2 deletion, which is consistent with the minimal expression of CAP2 in the lens epithelium. Given that changes in lens biomechanics can sometimes arise from alterations in fiber cell organization (Cheng et al., 2016b; Gu et al., 2019; Maddala et al., 2011), we next examined lens fiber cell organization in *CAP2^cKO^* lenses to determine whether the altered mechanics was accompanied by structural defects. Confocal imaging of lens cryosections showed that fiber cells in *CAP2^cKO^* lenses maintained their elongated shape and typical hexagonal packing in cross section (Fig. S4D). The characteristic alignment of fiber cells into radial cell columns and the formation of anterior and posterior sutures also appeared normal in cKO lenses (Fig. S4F,G). Minor deviations in fiber cell packing were observed in cKO lenses near the equator (Fig. S4D,E), but these changes were subtle and unlikely to account for the observed biomechanical phenotype, as other genetically modified lenses with extensive fiber cell hexagonal packing defects have no changes in biomechanical stiffness (Islam et al., 2023).

### Loss of CAP2 does not affect the ratio of F-actin to G-actin in lens fiber cells

**Fig. 3.**
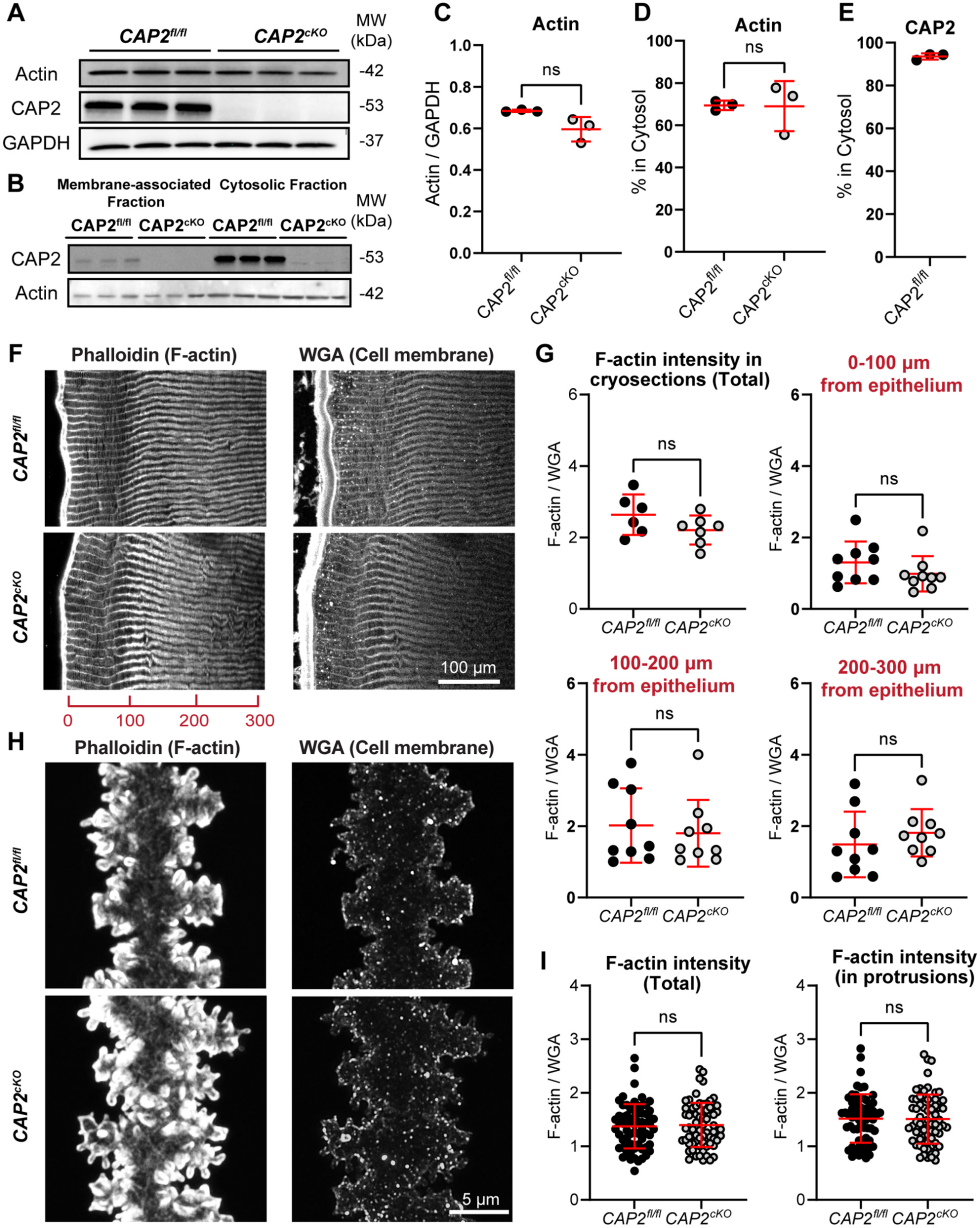
**The ratio between cytosolic and membrane-associated fractions of actin is similar in *CAP2^cKO^* and control lenses.** (A–E) Western blot of (A) lens total proteins and (B) cytoplasmic and membrane fractions. The CAP2 and GAPDH blots in A are the same as those shown in Fig. 1C, as they were derived from the same experiment. There is no change in (C) total actin levels or (D) the percentage of actin in the cytosol in mutant lenses. (E) Percentage of CAP2 in cytosol of control lenses. Plots reflect mean±s.d. of 3 independent protein samples per genotype. (G–I) Immunostaining of (F) lens cryosections and (H) single fiber cells with rhodamine phalloidin for F-actin and WGA for the cell membrane. Quantification of F-actin signal intensity normalized to WGA in (G) an entire cryosection and in three successive 100-μm regions from the epithelium, and in (I) protrusions and entire mature single fibers. Plots reflect mean±s.d. of 6–9 sections from six mice per genotype (G) and a total of 70 individual fibers from 9 mice per genotype (I). Scale bars: 100 μm (F), 5 μm (H). ns, not significant (two-tailed unpaired *t*-test).

Based on the ability of CAP2 to promote actin filament disassembly (Colpan et al., 2021; Kosmas et al., 2015; Peche et al., 2007), we hypothesized that deleting CAP2 in the lens might similarly alter actin polymerization and affect the proportion of F-actin relative to globular actin (the F-actin-to-G-actin ratio). To test this, we performed complementary biochemical (Fig. 3A–E) and immunostaining (Fig. 3F–I) approaches. The total actin level was not significantly different in *CAP2^cKO^* lenses, based on western blotting (Fig. 3A,B). Subcellular fractionation to isolate lens cytosol (supernatant) and membrane and cytoskeleton fractions (pellets), followed by western blotting, demonstrated that ∼69% of actin was present in the lens cytosol in the *CAP2^fl/fl^* lenses, similar to previous observations (Nowak et al., 2009; Woo and Fowler, 1994), with no increase in cytosolic levels of actin in the *CAP2^c^*^KO^ lenses (Fig. 3B,D). We also observed that ∼93% of CAP2 protein was cytosolic in control lenses (Fig. 3B,E), consistent with previous reports showing that CAP2 is distributed diffusely in the cytoplasm of mouse embryonic fibroblasts (Kepser et al., 2021) and cardiomyocytes (Colpan et al., 2021) in addition to its enrichment at the pointed ends of actin filaments in cardiomyocytes (Colpan et al., 2021). Therefore, we conclude that the absence of CAP2 does not significantly influence the overall F-actin-to-G-actin ratio within lens fiber cells.

We also investigated F-actin levels in control (*CAP2^fl/fl^*) and *CAP2^c^*^KO^ fiber cells by staining F-actin in lens cryosections with Rhodamine–phalloidin, using wheat germ agglutinin (WGA) as a marker for fiber cell membranes (Kistler et al., 1986). This showed that loss of CAP2 had no effect on the fluorescence intensity of F-actin with respect to WGA (Fig. 3F,G). Next, we considered whether the loss of CAP2 might lead to altered abundance of F-actin in different lens regions containing young, maturing and older lens fiber cells. However, the ratio of F-actin to WGA revealed no significant differences in F-actin fluorescence intensity between the *CAP2^cKO^* lens and the control in fiber cells at different depths (Fig. 3G).

We next considered the possibility that changes in actin polymerization in specific F-actin-rich structures, such as membrane protrusions, could underlie the increased stiffness. The F-actin-rich lens fiber cell membrane protrusions have been demonstrated to play a crucial role in determining the biomechanical properties of the lens (Cheng et al., 2018; Gokhin et al., 2012). Recently, we have shown that compressive loading of lenses induces significant curvature in cortical fiber bundles, accompanied by reversible distortion of membrane paddles and effacement of the small membrane protrusions (Cheheltani et al., 2025b). To investigate whether loss of CAP2 affects F-actin levels within these protrusions, we quantified F-actin signal intensity specifically within the membrane protrusion domains of single mature fiber cells (Fig. 3H,I). F-actin abundance within these protrusions was not significantly different from that of controls (Fig. 3I), suggesting that altered actin network organization, rather than filament polymerization and accumulation, could be the underlying mechanism causing the stiffer lens phenotype.

### Cofilin activation and localization are not altered in CAP2-deficient lenses

CAP2 promotes actin filament turnover in cooperation with ADF/cofilin family proteins. To determine whether CAP2 loss affects cofilin activity in the lens, we examined total cofilin-1 and Ser3-phosphorylated cofilin-1 (the inactive form), which are non-muscle isoforms of cofilin (Hotulainen et al., 2005), in control and CAP2 KO lenses by western blot analysis. Quantification revealed no significant differences in levels of total cofilin-1 or Ser3-phosphorylated cofilin (p-cofilin-1) (Fig. S5A,C).

We next examined the subcellular localization of cofilin-1 and p-cofilin-1 in isolated lens fiber cells by immunofluorescence. Both proteins exhibited comparable distributions relative to F-actin-rich membrane protrusions in control and CAP2 KO lenses, with no obvious genotype-dependent differences (Fig. S5B,D). Together, these data suggest that CAP2 deficiency does not measurably alter cofilin activation status or localization in lens fiber cells. This indicates that the effects of CAP2 loss on actin organization and lens biomechanics are unlikely to be mediated through changes in cofilin activity. Instead, CAP2 might influence actin network organization through alternative mechanisms, such as modulation of other actin-binding proteins or filament stability.

### Loss of CAP2 alters the levels of some actin-associated proteins in the lens

Prior studies have revealed that ABPs modulate lens biomechanics by altering cytoskeletal networks and F-actin-stabilizing proteins in fiber cells. For example, the absence of Tmod1 destabilizes the spectrin-F-actin network, leading to softer lenses (Cheng et al., 2016b; Gokhin et al., 2012). Likewise, Tpm3.5 deficiency results in dissociation of Tmod1 from F-actin at the membrane, as well as altered distribution of spectrin and reduced levels of T-plastin [also known as plastin-3 (PLS3)], leading to decreased lens stiffness under high loads (Cheng et al., 2018). Therefore, we hypothesized that the increased lens stiffness observed in the absence of CAP2 might stem from the opposite effect. In other words, loss of CAP2 could trigger the upregulation or redistribution of F-actin stabilizers and membrane-linkage proteins that reinforce the cortical cytoskeleton, thereby increasing tissue stiffness. To test this idea, we examined whether the absence of CAP2 alters the levels or localization of key F-actin stabilizing and crosslinking proteins in the lens.

**Fig. 4.**
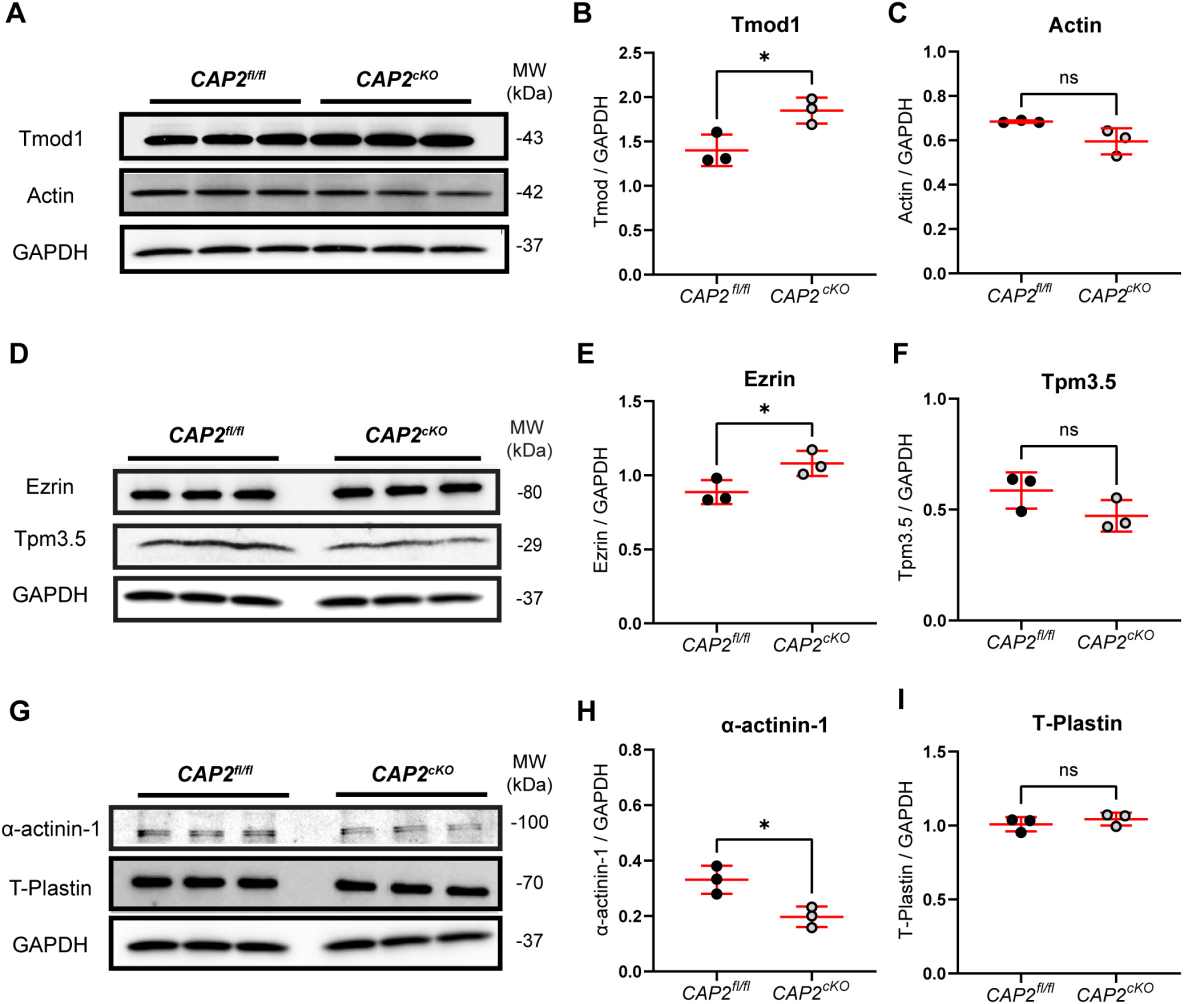
**Deletion of CAP2 alters levels of the actin-associated proteins α-actinin-1, Tmod1 and ezrin.** (A,D,G) Western blots of actin-associated proteins from 8-week-old whole lenses. The actin and GAPDH blots in A are the same as those shown in Fig. 3A, as they were derived from the same experiment. (B,C,E,F,H,I) Quantification of proteins from western blots, normalized to GAPDH. (B) The levels of Tmod1 and (E) ezrin are increased, while the levels of (C) actin and (F) Tpm3.5 are not significantly different in mutant lenses. (H) Total levels of α-actinin-1 protein are decreased in *CAP2^cKO^* lenses, while (I) T-plastin levels are unchanged. Plots show the mean±s.d. of 3 independent protein samples per genotype. *P<0.05; ns, not significant (two-tailed unpaired *t*-test).

First, to determine whether the absence of CAP2 affects the abundance of other cytoskeletal proteins, we assessed the total protein levels of key ABPs in whole-lens extracts from *CAP2^cKO^* and control mice. Western blot analysis revealed several notable changes (Fig. 4). The amount of Tmod1, a pointed-end capping protein that stabilizes actin filaments (Yamashiro et al., 2012), and ezrin, a membrane–cytoskeleton linker associated with F-actin-rich protrusions (Bretscher et al., 1997), were increased in CAP2-deficient lenses (Fig. 4A,B,D,E). In contrast, the amount of α-actinin-1, an anti-parallel F-actin cross-linker, was significantly reduced (Fig. 4G,H), whereas the level of T-plastin, which bundles parallel and anti-parallel F-actins (Mei et al., 2022) was unchanged between genotypes (Fig. 4G,I). Notably, the level of Tpm3.5 (Fig. 4D,F) was not significantly changed, suggesting that loss of CAP2 does not broadly perturb all actin cytoskeletal components.

### Actin-stabilizing protein associations with F-actin-rich protrusions are increased in *CAP2^cKO^* lenses

**Fig. 5.**
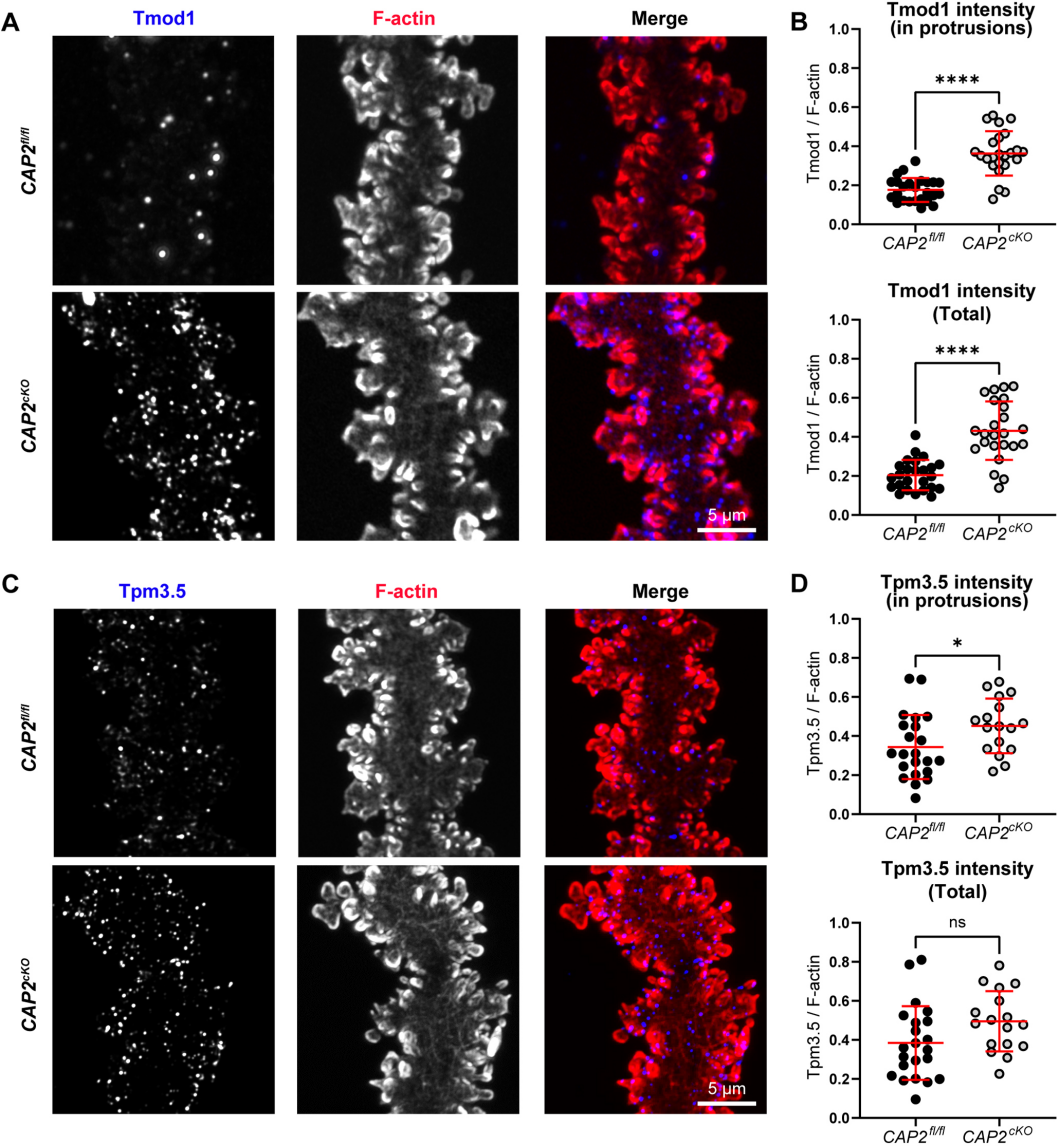
**Tmod1 and Tpm3.5 are increased in *CAP2^cKO^* lenses.** (A,C) Immunostaining of single mature fiber cells from 6-week-old *CAP2^cKO^* and *CAP2^fl/fl^* lenses for (A) Tmod1 (blue) and F-actin (red) and (C) Tpm3.5 (blue) and F-actin (red). Images are maximum intensity projections. Scale bars: 5 μm. (B,D) Quantification of fluorescence intensity of puncta relative to F-actin intensity in protrusions and across the whole fiber cell region. Plots represent the mean±s.d. of 20-30 individual fibers from six lenses of three different mice per genotype. *P<0.05; ****P<0.0001; ns, not significant (two-tailed unpaired *t*-test).

CAP2 regulates filament turnover at pointed ends (Colpan et al., 2021; Iwanski et al., 2021; Kotila et al., 2019); therefore, we investigated whether its deletion affects the distribution of Tmod1, the pointed-end capping protein in lens fiber cells. We first examined the fluorescence intensity of Tmod1 in lens equatorial cryosections at fiber cell regions extending from the epithelium to the inner fiber cells (Fig. S6A). Quantification of the Tmod1 intensity relative to F-actin intensity revealed that Tmod1 fluorescence intensity is significantly higher in *CAP2^cKO^* lenses compared to in control lenses, especially in the region spanning outer cortical and mature fiber cells (0–160 μm inwards from the epithelial cells) (Fig. S6B). Furthermore, closer examination of Tmod1 localization in individual mature fiber cells revealed that Tmod1 is associated with puncta in the F-actin-rich membrane protrusions of mature fiber cells in control and *CAP2^cKO^* lenses (Fig. 5A). We quantified the fluorescence intensity of the Tmod1 puncta in the protrusions and normalized it to F-actin intensity for individual mature fiber cells from control and *CAP2^cKO^* lenses. This revealed that in *CAP2^cKO^* lenses, that both the relative intensity of total Tmod1 and Tmod1 in the membrane protrusions of mature fibers were higher (Fig. 5A,B). The increased staining intensity of Tmod1 with respect to F-actin in *CAP2^cKO^* lenses suggests that loss of CAP2 leads to increases in Tmod1-capped F-actin in lens fiber cells.

As Tmod1 and Tpms are essential F-actin-stabilizing proteins that interact with one another, and previous studies have shown their importance in lens biomechanical properties (Cheng et al., 2018; Gokhin et al., 2012), we also evaluated the localization and abundance of the major Tpm isoform in lens fiber cells, Tpm3.5 (Cheng et al., 2018, 2016b). Consistent with enhanced F-actin stability, we detected an increase in Tpm3.5 localization associated with F-actin in or near the membrane protrusions of *CAP2^cKO^* mature fiber cells (Fig. 5C,D). This enrichment of Tpm3.5 with respect to F-actin in the absence of CAP2 implies that more actin filaments are decorated with Tpm3.5 and available for high-affinity capping by Tmod1 near the membrane of cKO fibers. Notably, although Tmod1 and Tpm3.5 exhibited distinct labeling patterns in single fiber cells, these images were obtained from different cells, and given the known heterogeneity in lens fiber cell architecture, such differences likely reflect variability in actin organization rather than mutually exclusive filament associations.

### CAP2 absence disrupts α-actinin-1-linked actin networks and increases T-plastin-linked F-actin bundles at fiber cell membranes

**Fig. 6.**
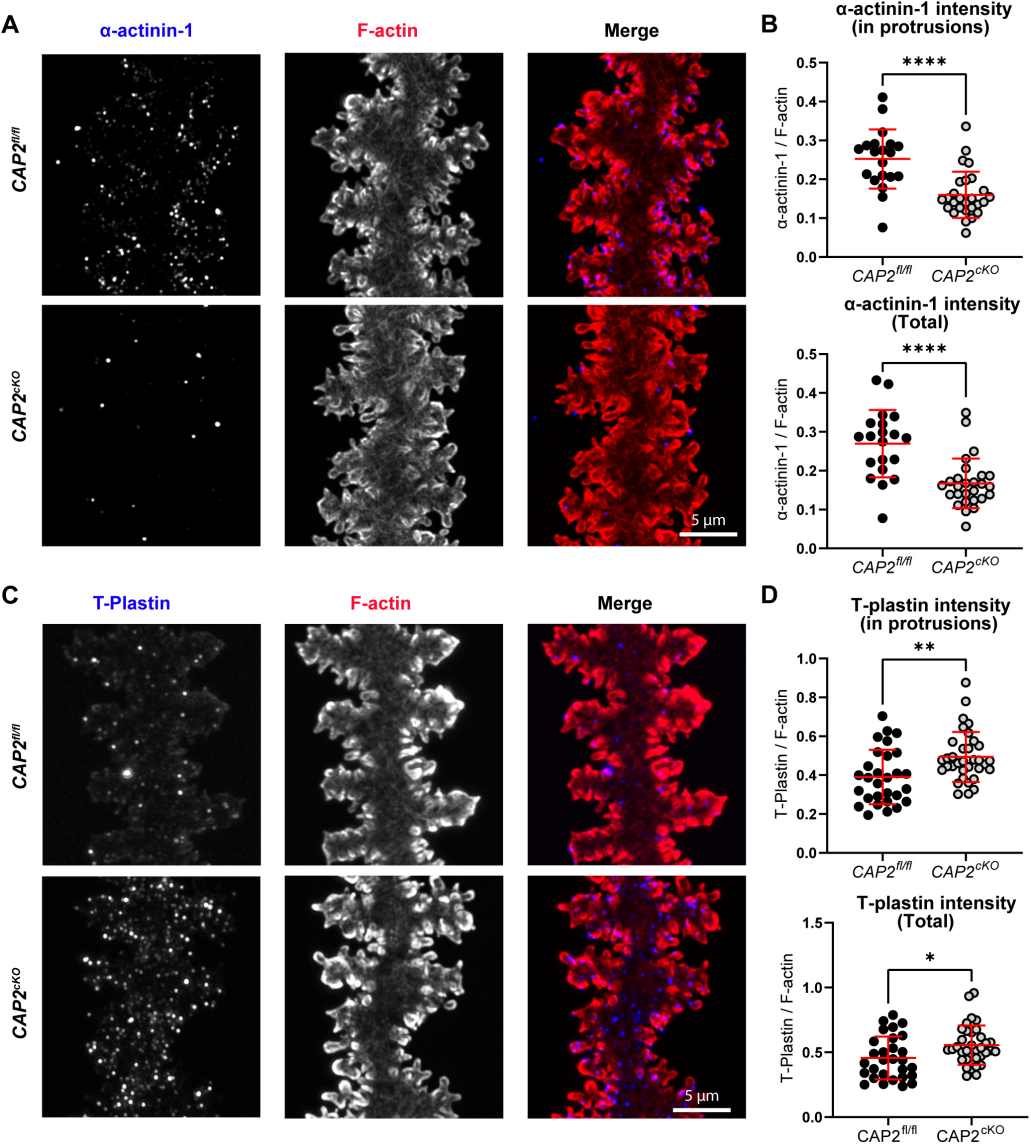
**The α-actinin-1-F-actin network is decreased while the T-plastin-F-actin network is increased in *CAP2^cKO^* lens fibers.** (A,C) Immunostaining of single mature fiber cells from 6-week-old *CAP2^fl/fl^* and *CAP2^cKO^* lenses for (A) α-actinin-1 (blue) and F-actin (red) and (C) T-plastin (blue) and F-actin (red). Images are maximum intensity projections. Scale bars: 5 μm. (B,D) Quantification of fluorescence intensity of puncta relative to F-actin intensity in protrusions and across the whole fiber cell region. Plots reflect the mean±s.d. of 20–30 individual fibers from six lenses of three different mice per genotype. *P<0.05; **P<0.01; ****P<0.0001 (two-tailed unpaired *t*-test).

In mouse lenses lacking F-actin-stabilizing proteins, such as Tmod1 or Tpm3.5, the F-actin organization in membrane protrusions is disrupted, with aberrant distributions of F-actin cross-linking proteins such as α-actinin-1 and T-plastin (Cheng et al., 2018, 2016b). For example, the softer Tpm3.5-deficient lenses exhibit increased prevalence of α-actinin-1 networks (Cheng et al., 2018), whereas softer Tmod1-KO lenses display expanded, nearly continuous α-actinin-1 localization along the fiber cell membrane, distinct from the punctate pattern observed in controls (Cheng et al., 2016b). Given these results, and the observed increase in lens stiffness in *CAP2^cKO^* lenses, we investigated whether decreases in α-actinin-1 might be observed in the mature single fiber cells without CAP2 (Fig. 6A,B). In control lens fiber cells, prominent α-actinin-1 puncta were found on the side, tips, and at the base of the small F-actin-rich membrane protrusions (Fig. 6A), whereas in the fiber cells from *CAP2^cKO^* lenses, the α-actinin-1 puncta intensity relative to F-actin are significantly reduced (Fig. 6A,B). A decrease in α-actinin-1 intensity relative to F-actin is also observed in younger, peripheral fiber cells, based on immunostaining of equatorial cryosections (Fig. S7). These results suggest that loss of CAP2 leads to reductions in α-actinin-1-linked F-actin networks in peripheral and mature fiber cells.

In contrast to α-actinin-1 networks, T-plastin networks were reduced in Tpm3.5-deficient lenses (Cheng et al., 2018). Therefore, we next examined T-plastin to determine whether its localization or expression was affected in CAP2-deficient lenses. In control lens fiber cells, sparse T-plastin puncta were associated with F-actin in membrane paddles and protrusions (Fig. 6C). In contrast, in lens fiber cells from *CAP2^cKO^* lenses, the T-plastin puncta appeared more abundant and their intensity relative to F-actin was significantly increased (Fig. 6C,D). This suggests that loss of CAP2 leads to expansion of F-actin bundles associated with T-plastin at the membrane protrusions of mature lens fibers.

### Loss of CAP2 leads to increased ezrin, whereas β2-spectrin localization remains unchanged

**Fig. 7.**
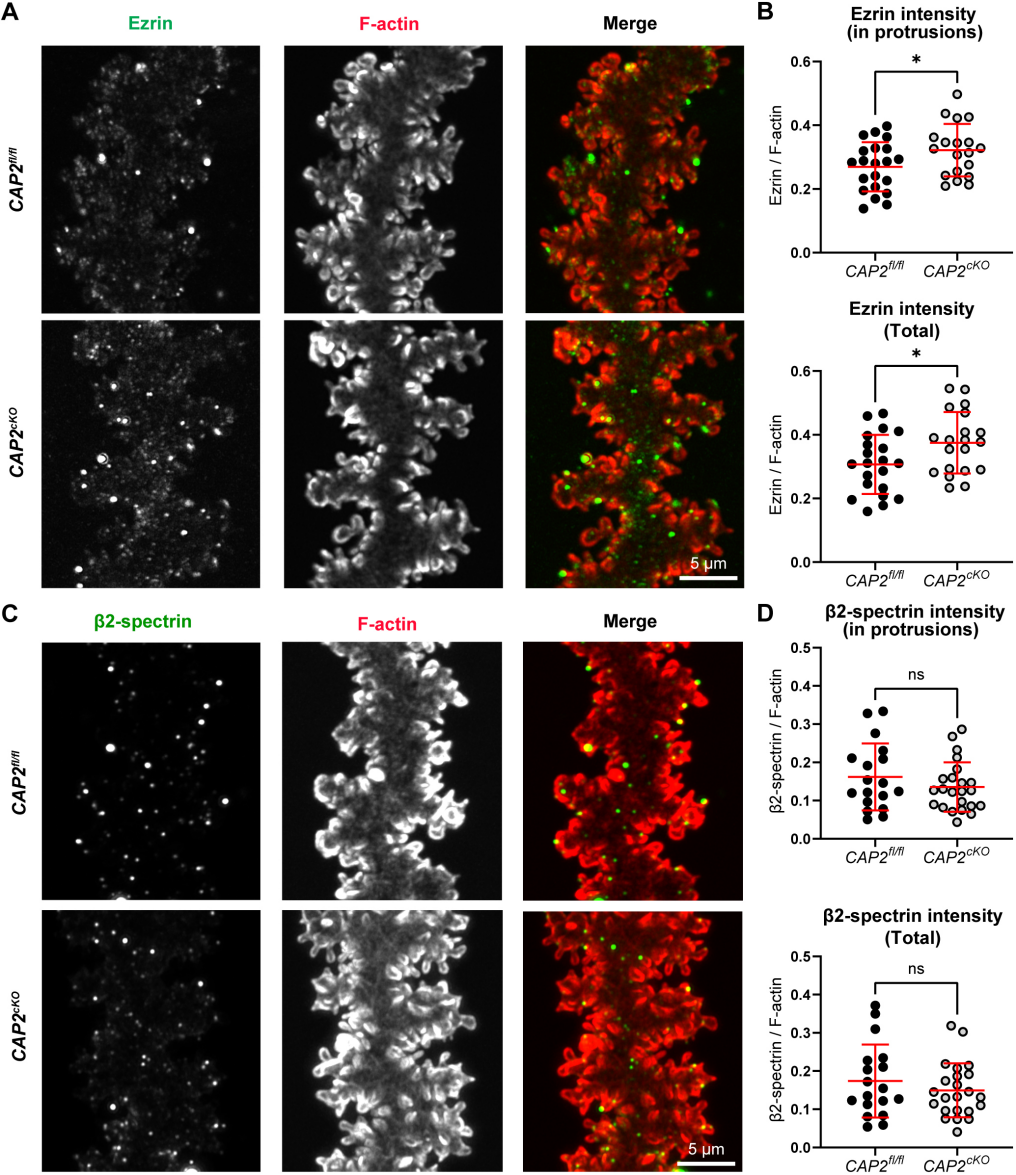
**Ezrin is increased in *CAP2^cKO^* lenses.** (A,C) Immunostaining of single mature fiber cells from 6-week-old *CAP2^fl/fl^* and *CAP2^cKO^* lenses for (A) ezrin (green) and F-actin (red) and (C) for β2-spectrin (green) and F-actin (red). Images are maximum intensity projections. Scale bars: 5 μm. (B,D) Quantification of fluorescence intensity of puncta relative to F-actin intensity in protrusions and across the whole fiber cell region. Plots represent the mean±s.d. of 20-30 individual fibers from six lenses of three different mice per genotype. *P<0.05; ns, not significant (two-tailed unpaired *t*-test).

As loss of Tmod1 leads to softer lenses and is associated with disrupted spectrin-actin networks (Cheng et al., 2016b), we hypothesized that an increased stiffness in CAP2-deficient lenses might involve the opposite effect, reinforcement of actin–membrane linkages. We therefore tested whether the levels or localization of key membrane–cytoskeleton linkers in the lens, such as ezrin and β2-spectrin, were altered in the absence of CAP2. Consistent with the increase in total ezrin levels in western blot of *CAP2^cKO^* lenses shown above (Fig. 4E), immunostaining showed an increase in ezrin puncta intensity in mature single lens fiber cells (Fig. 7A,B). In contrast, the localization and abundance of β2-spectrin, an essential scaffolding protein of the membrane-associated spectrin–actin cytoskeleton, remain unchanged in CAP2-deficient lenses (Fig. 7C,D). This suggests that the core spectrin–actin membrane skeleton is preserved, but specific membrane actin linkers, such as ezrin, might be selectively upregulated to compensate for the other alterations in F-actin network organization. Together, these protein-level changes reflect selective reorganization of the actin cytoskeletal network in CAP2-deficient lenses, particularly in the F-actin-enriched membrane protrusion regions of mature fiber cells.

## DISCUSSION

**Fig. 8.**
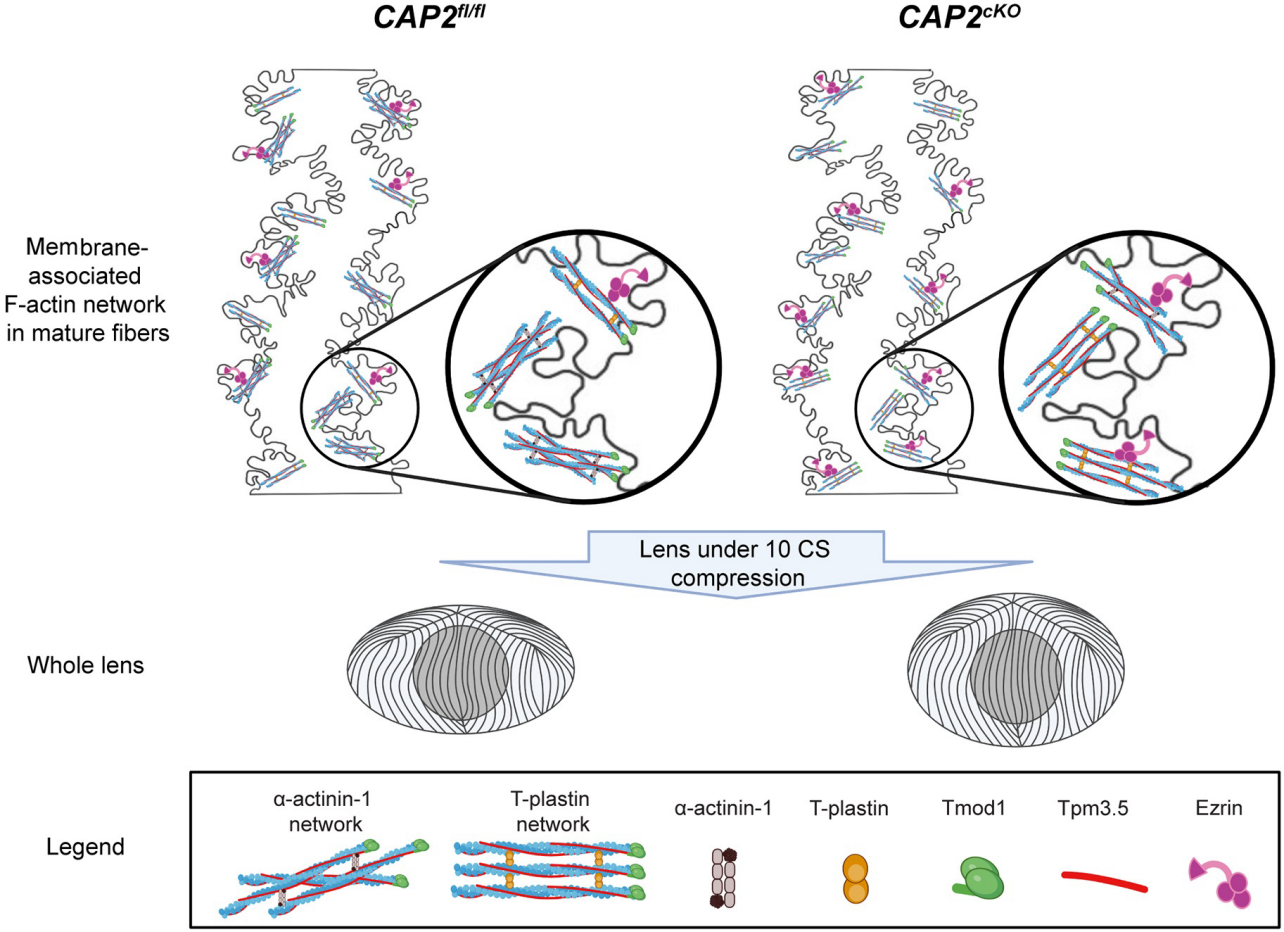
**Summary diagram illustrating the effects of *CAP2* deletion on lens biomechanics and membrane-associated F-actin networks.** Top: magnified diagrams of individual mature fiber cells depicting the organization of actin networks at the membrane. Black circles: close-up views of actin-binding protein interactions at the membrane. Loss of CAP2 leads to enhanced Tmod1 capping and increased association of actin filaments with Tpm3.5 and ezrin. Actin filaments are increasingly bundled by T-plastin, and there is a reduction in α-actinin-1-cross-linked filaments in *CAP2^cKO^* lens fibers. The arrow points to lenses from *CAP2^fl/fl^* (left) and *CAP2^cKO^* (right) mice under 10 coverslip (CS) compression, showing decreased axial strain in the *CAP2^cKO^* lens. Created in BioRender by Cheheltani, S., 2026. https://BioRender.com/cilm0ic. This figure was sublicensed under CC-BY 4.0 terms.

The biomechanical properties of the ocular lens are dependent on precise regulation of its cytoskeletal architecture, particularly within the F-actin-rich cortical regions of fiber cells (Cheng et al., 2018, 2017; Gokhin et al., 2012). F-actin stability, crosslinking and membrane tethering all contribute to the ability of the mouse lens to deform and recover in response to mechanical compression and load release (Cheng et al., 2018; Gokhin et al., 2012). Our current study identifies CAP2 as one of the key regulators of these processes, acting locally near the membrane of mature fiber cells to influence the structural organization of the F-actin cytoskeleton and mechanical properties of the lens (Fig. 8).

Using a lens-specific conditional knockout model, we found that CAP2 does not directly affect lens transparency and gross morphology but plays a role in modulating lens stiffness. In contrast, a previous study has reported microphthalmia in the global CAP2 KO mice (Field et al., 2015). This is likely due to early developmental defects arising from the loss of CAP2 in all ocular tissues, including the retina and optic cup (e.g. Fig. 3) (Harding et al., 2021; Verma and FitzPatrick, 2007). In our model, where CAP2 deletion is restricted to lens epithelial and fiber cells, the lenses displayed a normal shape and did not exhibit any signs of opacities. Yet, they were significantly stiffer under compressive load, suggesting that local cytoskeletal remodeling, rather than large-scale developmental and structural disruption, underlies the mechanical phenotype. The localization of CAP2 with membrane-associated F-actin in fiber cells points to a role in regulating F-actin turnover near the membrane, where actin network remodeling is likely essential for maintaining biomechanical plasticity.

Although the increase in lens stiffness observed in *CAP2^cKO^* mice is quantitatively modest, even small changes in lens mechanical properties can have meaningful functional consequences. The crystalline lens operates within a narrow biomechanical range, where subtle alterations in stiffness can influence lens deformation, internal strain distribution and optical organization (Kasthurirangan et al., 2008; Khan et al., 2018; Wang and Pierscionek, 2019). Although mice lack classical accommodative ability, lens stiffness in the mouse eye remains tightly linked to fiber cell compaction and optical quality, making the mouse lens a good model for probing cytoskeletal regulation of tissue mechanics independent of accommodation (Cheng et al., 2019; Rodriguez et al., 2023).

### CAP2 regulates mouse lens stiffness through local remodeling of actin networks

In comparison to previous studies, which have shown that deletion or reduction of F-actin-stabilizing proteins such as Tmod1 or Tpm3.5 results in decreased lens stiffness in the mouse (Cheng et al., 2018, 2016b; Gokhin et al., 2012; Parreno et al., 2020), the deletion of CAP2 resulted in increased lens stiffness, supporting the idea that CAP2 acts antagonistically to F-actin-stabilizing factors. In cardiomyocytes and other cells, CAP2 enhances the turnover of ADF/cofilin-bound F-actin by targeting filament pointed ends for disassembly (Colpan et al., 2021; Kumar et al., 2016; Makkonen et al., 2013; Quintero-Monzon et al., 2009). In contrast, Tmods and Tpms cap and stabilize F-actin pointed ends, respectively (Cheng et al., 2016b; Kostyukova, 2008; Weber et al., 1994), a relationship shown to oppose CAP-mediated turnover (Colpan et al., 2021; Iwanski et al., 2021). In *CAP2^cKO^* lenses, we observed an increase in both Tmod1 and Tpm3.5 localization within the F-actin-rich membrane protrusions of mature fibers, suggesting that in the absence of CAP2, filaments are stabilized by increased pointed end capping by Tmod1. Additionally, increased Tpm3.5 at the F-actin-rich protrusions of *CAP2^cKO^* lenses supports the view that filament turnover is reduced, likely leading to increased rigidity of the actin networks at the membrane (Fig. 8).

Notably, the actin filament stabilization is accompanied by selective remodeling of cross-linked F-actin networks, which can further contribute to lens stiffness in the absence of CAP2. α-actinin-1-associated puncta are reduced in cKO lenses, whereas T-plastin-associated puncta are increased. α-Actinin-1 crosslinks F-actin into loose, contractile arrays of anti-parallel filaments, whereas T-plastin bundles actin into tightly-packed, parallel and anti-parallel filaments, which form stiffer networks (Bretscher, 1981; Matsudaira, 1994; Winkelman et al., 2016). Although α-actinin-1 and T-plastin both function as F-actin-bundling proteins, they organize filament networks with distinct geometries and are often mutually exclusive (Cheng et al., 2018; Yamashiro-Matsumura and Matsumura, 1986). This suggests that the shift from α-actinin-1 to T-plastin-associated F-actin networks could contribute to the increased stiffness by decreasing cytoskeletal flexibility and promoting structural compaction (Yamashiro-Matsumura and Matsumura, 1986). Interestingly, another membrane-associated cytoskeletal component, β2-spectrin, remained unchanged, suggesting that CAP2 loss selectively alters some F-actin networks without disrupting the underlying membrane-associated scaffold (Cheng et al., 2017; Leterrier and Pullarkat, 2022). This suggests a model in which CAP2 and Tmod1 exist in a local regulatory balance where CAP2 facilitates pointed-end turnover, while Tmod1 caps and stabilizes the ends, as proposed for thin filaments in striated muscle and during cardiac myofibrillogenesis (Iwanski et al., 2021). When CAP2 is removed, this balance shifts toward the stabilization of filaments and the rearrangement of filament networks, ultimately leading to altered fiber cell and lens mechanics, as depicted in Fig. 8.

The abundant actin cytoskeleton within fiber cell membrane interdigitations is supported by membrane–cytoskeletal linkers, including components of the ezrin, periplakin, periaxin and desmoyokin (EPPD) cell junctional complex, which also includes spectrin (Audette et al., 2017; Blankenship et al., 2007; Cheng et al., 2017; Gonen et al., 2004; Lindsey Rose et al., 2006; Rao and Maddala, 2006; Straub et al., 2003). Ezrin, a member of the ezrin-radixin-moesin (ERM) family, links F-actin to the plasma membrane and regulates cell shape and cortical stiffness (Bagchi et al., 2004; Ivetic and Ridley, 2004; Louvet-Vallée, 2000; Rao et al., 2008). In its active form, ezrin undergoes phosphorylation-driven conformational changes that enable its interaction with both F-actin and membrane proteins, facilitating the formation of membrane protrusions, supporting cortical tension, and increasing cytoskeletal stiffness in various cell types (Viswanatha et al., 2013; Zhang et al., 2020). In *CAP2^cKO^* lenses, we observed increased total ezrin protein levels and enhanced ezrin localization at the membrane of mature fiber cells, particularly at F-actin-rich protrusions. These findings suggest that, in addition to filament stabilization and altered crosslinking, upregulation of ezrin-mediated membrane-actin tethering potentially leading to increased cortical stiffness might further contribute to the stiffer biomechanical phenotype of CAP2 KO lenses. Although we detected an increase in total ezrin, further work is needed to determine whether the active form is enriched in the KO lenses. If so, activated ezrin could further reinforce membrane–cytoskeletal interactions, contributing to increased resistance to deformation.

Although CAP2 appears to regulate lens biomechanics through changes in F-actin-stabilizing and cross-linking proteins, we cannot exclude contributions from other actin cytoskeletal regulators. One potential candidate is non-muscle myosin II (NM2), a motor protein that assembles into bipolar filaments, which interact with F-actin, generating contractile tension and contributing to actin network organization in many cell types. However, a recent study by Islam et al. (2023) has demonstrated that disruption of NMIIA assembly in the lens epithelium and differentiating fiber cells altered epithelial cell alignment and fiber cell hexagonal packing, yet did not affect lens size, shape, transparency or stiffness (Islam et al., 2024, 2023). These findings suggest that perturbations of NM2 alone are insufficient to modify whole-lens biomechanical properties. Accordingly, although our data implicate CAP2 in modulating the organization and levels of selected actin-binding proteins, its biomechanical effects might also involve more complex remodeling of distinct actin filament subpopulations or interactions with other regulatory pathways that may involve NM2.

### Implications for Cap2 functions in cell and tissue mechanics and motility

Beyond the lens, CAP2-mediated actin turnover is broadly required for the formation of dynamic protrusions and efficient migration (Rust et al., 2020). Motility defects are observed in fibroblasts of CAP2 KO mice, where the loss of CAP2 results in increased focal adhesions, extended protrusions and higher F-actin content, consistent with stabilization of peripheral actin networks, without changes in total actin. (Kosmas et al., 2015). Similarly, primary keratinocytes from CAP2 KO mice migrate more slowly in scratch-wound assays, showing a delayed closure of the scratch compared to wild-type cells (Kosmas et al., 2015). In carcinoma models, CAP2 colocalizes with F-actin at the leading edge of lamellipodia, and its depletion impairs lamellipodial extension and motility (Effendi et al., 2013). Conversely, endoplasmic reticulum (ER)-stress-driven upregulation of CAP2 enhances migration and invasion (Yoon et al., 2021). Although these studies did not directly measure other ABPs, they showed increased F-actin and altered bundling at the cell periphery without changes in total actin (Kosmas et al., 2015), mirroring the redistribution of F-actin stabilizers without overall F-actin changes that we observed in the CAP2 KO lens. Thus, although lens fiber cells are non-motile, CAP2 regulation of their F-actin networks may resemble that of CAP2 regulation in motile cells.

Lens fiber cell paddles and protrusions in the cell–cell interlocking interdigitations are enriched with F-actin that is stabilized by Tpm3.5 and Tmod1, cross-linked by T-plastin, and anchored to the cell membrane by ERM proteins (Cheng et al., 2018; Gokhin et al., 2012; Straub et al., 2003). They also maintain the fiber-to-fiber cell interactions mediated by cell adhesion molecules, such as cadherins and AQP0 (Bassnett et al., 1999; Wang and Schey, 2011). In lens fibers, CAP2 deletion coincides with increased Tmod1, Tpm3.5, T-plastin and ezrin, suggesting that CAP2 normally limits actin stabilization at fiber–fiber cell interfaces. This observation might reflect a broader role for CAP2 in coordinating actin monomer recycling and ABP interactions, rather than a simple defect in filament turnover. In its absence, the actin membrane network becomes more stabilized and rigidly bundled, likely stiffening the paddles and interdigitations and, consequently, the entire lens. To our knowledge, this is the first demonstration that CAP2 regulates cell and tissue biomechanics in a non-muscle system by modulating F-actin-associated proteins, linking molecular control of actin turnover to tissue-level mechanical behavior in an intact vertebrate organ.

## MATERIALS AND METHODS

### Generation of lens-specific *CAP2^cKO^* mice

All animal procedures were conducted in adherence to the ARVO Statement for the Use of Animals in Ophthalmic and Vision Research and performed in accordance with approved animal protocols from the Institutional Animal Care and Use Committee guidelines at the University of Delaware. The *CAP2^LoxP^* mouse was obtained from Dr Jeffrey Field at the University of Pennsylvania, USA (Field et al., 2015). The targeted mouse was created by the European Conditional Mouse Consortium (EUCOMM), which provided the targeting construct and embryonic stem cell (ESC) clones (*CAP2^tm1a(EUCOMM^*^)^), which Dr Field crossed with the actin-FLP mouse to excise the FRT sites, *LacZ* and *neo* cassettes. This process generated the *tm1c* allele, restoring CAP2 expression (Kanuri et al., 2021; Skarnes et al., 2011). This transgenic mouse harbors the CAP2 gene with two LoxP (locus of X-over P1) sites flanking exon 3 (*CAP2^LoxP^* or *CAP2^fl/fl^*) (Field et al., 2015). Exon 3 is crucial for CAP2 function as it encodes the HFD domain responsible for F-actin-severing activity. Subsequently, we bred the *CAP2^fl/fl^* mice with *MLR10-Cre* mice [STOCK *Tg (Cryaa-Cre*)10Mlr/J, Jackson Labs] obtained from Dr Melinda Duncan at the University of Delaware, USA, resulting in the generation of *CAP2^fl/fl Tg (Cryaa-Cre)10Mlr^* (referred to as *CAP2^cKO^*) transgenic mice with lens-specific CAP2 deletion. The *MLR10-Cre* mouse features a deliberate insertion of a 20-bp Pax6 consensus-binding site within the transgenic αA-crystallin promoter, enabling CRE transgene expression in both lens fiber cells and epithelium (Zhao et al., 2004). CRE expression from these promoters initiates in the lens vesicle at E10.5 in *MLR10-Cre*, leading to the deletion of floxed exon 3 in the lens, thereby knocking out CAP2 in both lens epithelial and fiber cells (Field et al., 2015; Lam et al., 2019). Throughout this study, 6–8-week-old mice were used, with *CAP2^fl/fl^* mice serving as littermate controls. All mice were genotyped through TransnetYX (Cordova, TN) by semi-quantitative real-time PCR using primer pairs targeting the *CAP2^fl/fl^*, *CAP2^flfl; Tg (Cryaa-Cre)10Mlr^* and CRE alleles.

### Lens biomechanical testing and morphometrics

Lenses from 8-week-old mice were used for compression assay and morphometric measurements. Freshly dissected lenses were transferred to a custom-made chamber filled with 1× phosphate-buffered saline (PBS) [diluted 1:10 from Gibco PBS (10×), pH 7.4, 70011044, Thermo Fisher Scientific], and top-view images were acquired using an Olympus SZ11 dissecting microscope attached to a digital camera. To test the biomechanical properties of the lens, we conducted a coverslip compression assay as previously described (Cheng et al., 2016a). A series of ten 18×18 glass coverslips (∼114.8 mg each) (12542A, Fisherbrand, Pittsburgh, PA, USA) were sequentially placed on top of freshly dissected lenses, positioned in a divot inside the chamber. Side-view images of the lenses were captured using a 45° angled mirror. The equatorial and axial diameters of the lenses before and after each loading step were measured using FIJI software. Axial and equatorial strains were calculated from: ε=(d−d0)/d0, where ε is strain, d denotes the axial or equatorial diameter at a given load, and d0 represents the corresponding axial or equatorial diameter at zero load (Cheng et al., 2016a; Fudge et al., 2011; Gokhin et al., 2012). Lens volume was calculated from: volume=4/3×π×*r*_E_²×*r*_A_, where *r*_E_ is the equatorial radius and *r*_A_ is the axial radius (Sindhu Kumari et al., 2015). The lens aspect ratio was determined by dividing the equatorial diameter by the axial diameter. To evaluate the morphology of the lens nucleus, we meticulously removed peripheral fiber cells by gently rolling the lens between gloved fingertips, revealing a densely compacted central region known as the lens nucleus. Nuclear volume was calculated from volume=4/3×π×*r*_N_³, where *r*_N_ is the radius of the lens nucleus. The nuclear fraction is the ratio of the nuclear volume to the total lens volume (Gokhin et al., 2012).

### RNA isolation and RT-qPCR

Two lenses of each mouse were pooled and homogenized in 100 μL of TRIzol reagent (15596026, Thermo Fisher Scientific) for RNA isolation. The amount of RNA was measured by determining the absorbance at 260 nm (A_260_), and an equal amount of RNA from each lens sample was used for cDNA synthesis. Reverse transcription and cDNA synthesis were performed using the Superscript^TM^ III First-Strand Synthesis System kit (18080051, Thermo Fisher Scientific). RT-qPCR was performed on an equal amount of cDNA from each sample in a 20 μl volume containing TaqMan™ Fast Advanced Master Mix for qPCR (4444557, Thermo Fisher Scientific), TaqMan probes for Cap1 (Mm00482950_m1, Thermo Fisher Scientific), Cap2 spanning exons 2 and 3 (Mm00482639_m1, Thermo Fisher Scientific), and GAPDH (Mm99999915_g1, Thermo Fisher Scientific) as the housekeeping gene for normalization. Three lens pairs from each genotype were used for each experiment.

### Western blotting

Western blots were performed on lenses isolated from 8-week-old mice, as previously described (Cheng et al., 2018). Freshly dissected lenses were stored at −80°C until homogenization. Two lenses of each mouse were pooled into a single protein sample. Lens pairs were homogenized on ice in 250 μl of lens homogenization buffer (20 mM Tris-HCl pH 7.4 at 4°C, 100 mM NaCl, 1 mM MgCl_2_, 2 mM EGTA and 10 mM NaF with 1 mM DTT), 1:100 protease inhibitor cocktail (P8430, Sigma-Aldrich) and 1:1000 phosphatase inhibitor (78420, Thermo Fisher Scientific) per 10 mg of lens wet weight. To separate cytosolic and membrane-associated proteins, the homogenate was centrifuged at 21,130 ***g*** for 20 min at 4°C. The supernatant, representing the cytosolic fraction, was transferred to a new tube. The pellet, containing membrane-associated proteins, was washed twice with homogenization buffer and centrifuged at 21,130 ***g*** for 10 min between washes, then resuspended in homogenization buffer. The total lysates, or supernatants and resuspended pellets, were then diluted 1:1 with 2× Laemmli sample buffer (1610737, Bio-Rad Laboratories, Hercules, CA, USA). Samples were briefly sonicated with a Q55 Sonicator (Qsonica, Newtown, CT, USA) and boiled for 5 min. Proteins were separated by electrophoresis on 4–20% linear gradient SDS-PAGE mini-gels (XP04205BOX, Thermo Fisher Scientific) and transferred to nitrocellulose membranes (10600011, Amersham Protran, Slough, UK) at 150 V in 1× transfer buffer (25 mM Tris-HCl pH 8.3, 192 mM glycine in ddH_2_O) with 20% methanol in a trans-blot tank (Bio-Rad) at 4°C for 1 h. Membranes were stained with Ponceau S (09189, Fluka BioChemica, Mexico City, Mexico) to visualize total protein loading and gently washed with ddH_2_O. The blots were scanned with a Bio-Rad ChemiDoc MP to reveal total protein levels in each lane and blocked with 5% BSA in 1× PBS for 1 h at room temperature. The blots were then incubated with primary antibodies diluted in 5% BSA+0.1% Triton X-100 in PBS overnight at 4°C with gentle rocking.

The primary antibodies used for western blotting were anti-actin (C4, 1:20,000, Millipore, Burlington, MA, USA), anti-CAP2 (15865-1-AP, 1:1000 Proteintech), anti-α-actinin-1 (non-sarcomeric, Actn1, A5044, 1:1000, Sigma-Aldrich), anti-T-plastin (ab137585, 1:1000, Abcam), anti-Tmod1 (NBP2-00955, 1:1000, Novus Biologicals), anti-Tpm3.5 (CH1, 0.5 ug/ml, Developmental Studies Hybridoma Bank, Iowa City, IA, USA), anti-ezrin (E8897, 1:1000, Sigma-Aldrich), anti-GAPDH (NB300-221, 1:1000, Novus Biologicals), anti-cofilin-1 (MA5-17275, 1:1000, Thermo Fisher Scientific) and anti-p-cofilin-1 (3313, 1:1000, Cell Signaling Technology). The secondary antibodies (1:20,000) were IRDye-800CW-conjugated goat anti-rabbit-IgG (926-32211, LI-COR, Lincoln, NE, USA), IRDye-680LT-conjugated goat anti-mouse-IgG (926-68020, LI-COR) or IRDye-680RD-conjugated goat anti-mouse IgM (μ chain specific) (925-68180, LI-COR). After incubation with secondary antibodies, the blots were washed with 1× Tris-buffered saline with 0.1% Tween 20 detergent (TBST) (4×5 min per wash). Antibodies and other reagents are also listed in Table S1. The band intensities of the blot were quantified using FIJI software with background subtraction and normalized to total protein (Ponceau S staining) or GAPDH. The percentage of each protein in the cytosol was calculated by dividing the cytosol band intensity by the intensity of the cytosol band plus the membrane band. Three lens pairs from each genotype were used for each experiment. Uncropped images of blots from this paper are shown in Fig. S9.

### Immunostaining of frozen sections

To prepare frozen sections of the eye, a small opening was made at the corneal-scleral junction of freshly enucleated eyeballs to allow penetration of fixative (Cheng et al., 2016b). Eyeballs were fixed for 4 h at 4°C in 1% paraformaldehyde (PFA) in PBS, prepared fresh from a 16% stock (15710, Electron Microscopy Sciences, Hatfield, Pennsylvania, USA). Samples were subsequently washed in cold PBS three times and cryoprotected in 30% sucrose for ∼2 h, until sinking, and then embedded in OCT medium (Sakura Finetek, Torrance, CA, USA) in cross or sagittal-section orientation in blocks. Frozen sections (12 μm thick) were collected with a Leica CM3050 cryostat and stored at −20°C. Sections were briefly rehydrated with PBST (1× PBS, 0.01% Triton X-100) and permeabilized and blocked in blocking buffer (3% BSA, 3% goat serum, and 0.3% Triton X-100 in 1× PBS) for 1 h before incubation with the primary antibody. Lens sections were labeled with primary antibody at 1:100 in blocking buffer overnight at 4°C, washed in PBST (3×5 min) and labeled for 90 min with secondary antibody along with Rhodamine–phalloidin (R415, 220 nM, Thermo Fisher Scientific), WGA (Alexa Fluor™ 488 Conjugate, W11261, 1:500, Life Technologies) and Hoechst 33342 (62249, 1:1000 dilution, Thermo Fisher Scientific). Secondary antibodies IgG (1:200 dilution) were Alexa-Fluor-647-conjugated goat anti-mouse IgG (A21236, Thermo Fisher Scientific) or Alexa-Fluor-647-conjugated goat anti-rabbit. Sections were washed in PBST (4×5 min), mounted with ProLong Gold antifade reagent (P36934, Thermo Fisher Scientific) and sealed with clear nail polish. Imaging was performed using a Zeiss LSM880 confocal microscope (5× objective, NA 0.16, 20× objective, NA 0.8, or 63× oil immersion objective, NA 1.4).

### Immunostaining of single fiber cells

Single fiber cells were prepared as previously described (Vu and Cheng, 2023). Briefly, lenses were dissected from freshly enucleated eyeballs, and the lens capsule was removed using sharp tweezers. Decapsulated lenses were fixed in 1% PFA overnight at 4°C with gentle rocking. Fiber masses were then cut into quarters in sagittal orientation using a scalpel and post-fixed in 1% PFA for 15 min with gentle rocking at room temperature. Lens quarters were then washed in 1× PBS (2×5 min each) and blocked for 1 h in PBS containing 3% BSA, 3% goat serum, and 0.3% Triton X-100. Quarters were then incubated overnight at 4°C with gentle rocking in a 1:50 dilution of the primary antibody. After washing the lens quarters with PBST (3×10 min), they were labeled with secondary antibodies (1:100), Rhodamine–phalloidin (1:100) and WGA (1:100) for 3 h at room temperature. After incubation with secondary antibodies, the lens quarters were washed again (3×10 min). Then, fiber cells were separated from each other using two tweezers on a drop of ProLong Gold antifade reagent. A coverslip was then sealed onto each slide. *Z*-stacks of single fiber cells were acquired at a digital zoom of 3.0 and a z-step of 0.17 μm using a Zeiss LSM880 microscope (63× oil immersion objective, NA 1.4, AiryScan Super-resolution).

### Whole-mount staining and capsule thickness measurement

Whole fixed lenses were fluorescently labeled with Rhodamine–phalloidin (1:300), WGA (1:250) and Hoechst 33342 (1:500), as previously described (Emin et al., 2024). Each lens was positioned on a glass-bottom dish with its anterior surface facing the inverted microscope objective to visualize the anterior capsule. Z-stacks of the capsule were acquired with a digital zoom of 1.0 with *z*-step size of 0.3 μm using a Zeiss LSM880 confocal microscope with a 40× oil immersion objective, NA 1.3. Images were processed in Zeiss Zen Software (Blue 3.7) to generate *x-z* projections, and capsule thickness was measured as described previously (Cheheltani et al., 2025a; Parreno et al., 2018). Briefly, the distance between the outer surface of the capsule stained with WGA and the membrane of the underlying actin cytoskeleton labeled with rhodamine phalloidin was measured using line scan analysis in FIJI software (Fig. S4A–C).

To visualize the anterior and posterior sutures, lenses were positioned with either the anterior or posterior surface facing the objective, and images were acquired using the 20× objective in the rhodamine channel to detect F-actin labeling.

### Image analysis

To assess fiber cell organization in *CAP2^cKO^* and *CAP2^fl/fl^* control lenses, we quantified regions of disordered fiber cell packing in equatorial cryosections labeled with F-actin, as previously described (Islam et al., 2024, 2023). Briefly, single optical sections captured at 20× magnification were analyzed in FIJI software by manually outlining areas of disrupted fiber organization. The area of each disordered region was measured, and the percentage disorder was calculated by dividing the total disordered area by the area of the region of interest. Three sections from at least three different mice per genotype were analyzed for this quantification. The person performing analysis was aware of the experimental conditions; however, quantification was performed by objectively outlining the disordered regions based on morphological criteria.

To determine the fluorescence intensity in lens cryosections, the split channel function in the FIJI software was used to separate the red and green channels. The same area of the fiber cell was then outlined, and the mean fluorescence intensity was measured. The mean fluorescence intensity of Rhodamine–phalloidin was normalized to WGA fluorescence intensity. To assess F-actin intensity at different fiber cell depths, regions spanning 100 μm intervals from the epithelium (e.g. 0–100 μm, 100–200 μm and 200–300 μm) were outlined in both channels. To quantify the fluorescence intensity of ABPs in lens cryosections, the signal intensity of the protein of interest was normalized to Rhodamine–phalloidin. Six to seven different sections from three mice per genotype were used.

To determine the fluorescence intensity of individual ABPs in single fiber cells, *Z*-stack confocal images were further analyzed in Zen 3.7 microscopy software (Zeiss). Maximum intensity projections from *Z*-stacks were exported into FIJI software, and the mean fluorescence intensity in the membrane protrusions of mature fiber cells was measured by outlining the bright rhodamine-phalloidin-stained protrusion area of each single fiber cell with the FIJI freehand tool (Fig. S8). For each cell, the mean fluorescence intensity of each protein in membrane protrusions was divided by the mean rhodamine–phalloidin intensity measured for that same cell, yielding a normalized intensity. Data represent means determined for 20–30 single fiber cells from three different mice per genotype.

### Statistical analysis

Statistical significance was determined using a two-tailed unpaired Student's *t*-test or two-way ANOVA with Šídák's multiple comparisons test, and mean and s.d. were calculated and plotted using GraphPad Prism 9. A *P*-value less than or equal to 0.05 was considered statistically significant.

## Supplementary Material



10.1242/joces.264491_sup1 Supplementary information
