# Supplementary material for: Loss of cyclase-associated protein 2 alters actin cytoskeleton organization and biomechanical properties of the ocular lens
Source: J Cell Sci. 2026 May 26;139(10):jcs264491. doi: 10.1242/jcs.264491 (PMC13286373; doi:10.1242/jcs.264491)
Supplement: Supplementary information [file joces-139-264491-s1.pdf]

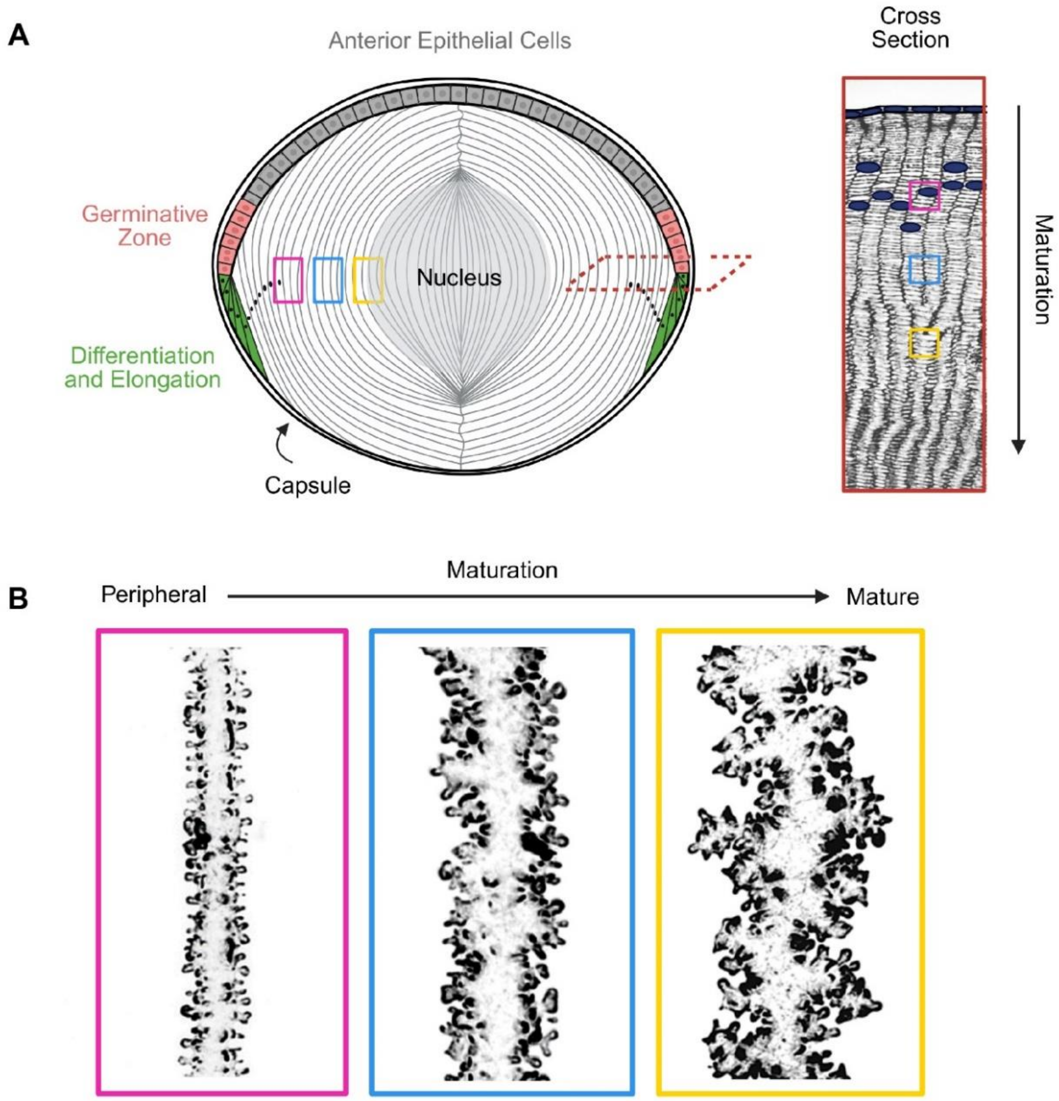

Fig. S1. Lens anatomy diagrams. (A) A sagittal section of the lens shows an anterior region of epithelial cells (gray) surrounded by a bulk mass of lens fiber cells (white). The compacted primary fiber cells, known as the nucleus, are located in the center of the lens (shaded area). The entire lens is encapsulated by a basement membrane called the capsule (Created in BioRender; Cheheltani, S. (2026) https://BioRender.com/j3cx4b3). The red dotted line shows a region where the cross-section in the red box is associated with. (B) Representative image of fiber cells at different stages of maturation. Peripheral fiber cells have small protrusions along their membrane (pink box). During maturation, cortical fiber cells develop larger protrusions along the short sides (blue box), and mature fiber cells from the organelle-free zone have large paddle domains decorated by small protrusions (yellow box).

Journal of Cell Science • Supplementary information

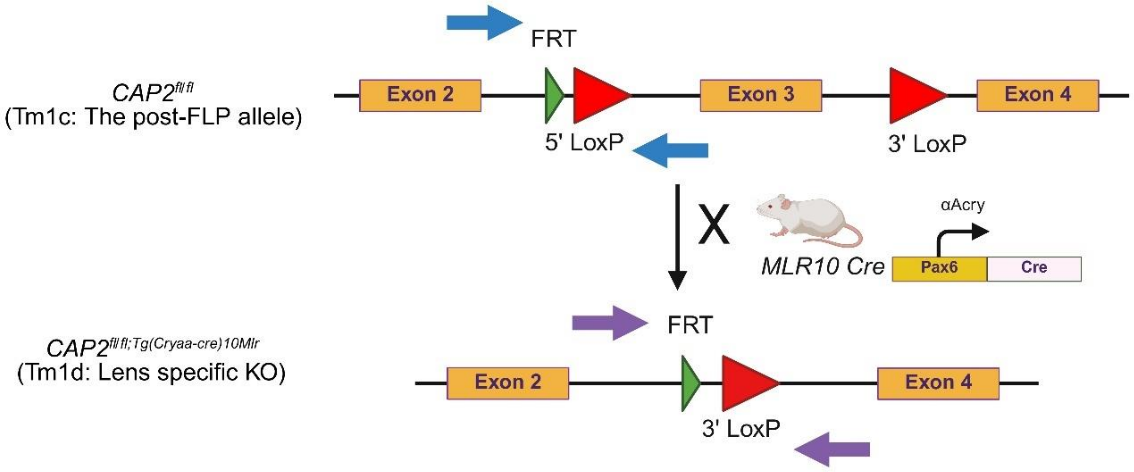

Fig. S2. Diagram of the strategy to create the lens-specific CAP2 KO mouse. Mice homozygotes for the *CAP2^fl/fl^* allele were crossed with *MLR10-Cre* transgenic mice to generate lens-specific *CAP2* KO mice. The blue arrows indicate the location of the reverse and forward primers for genotyping the *CAP2^fl/fl^* allele, which is used as a littermate control, and the purple arrows indicate the location of the primers used to genotype the *CAP2^cKO^* allele (Created in BioRender. Cheheltani, S. (2026) https://BioRender.com/9kl05s5). The primer design and genotyping were performed by TransnetYX using real-time PCR.

Journal of Cell Science • Supplementary information

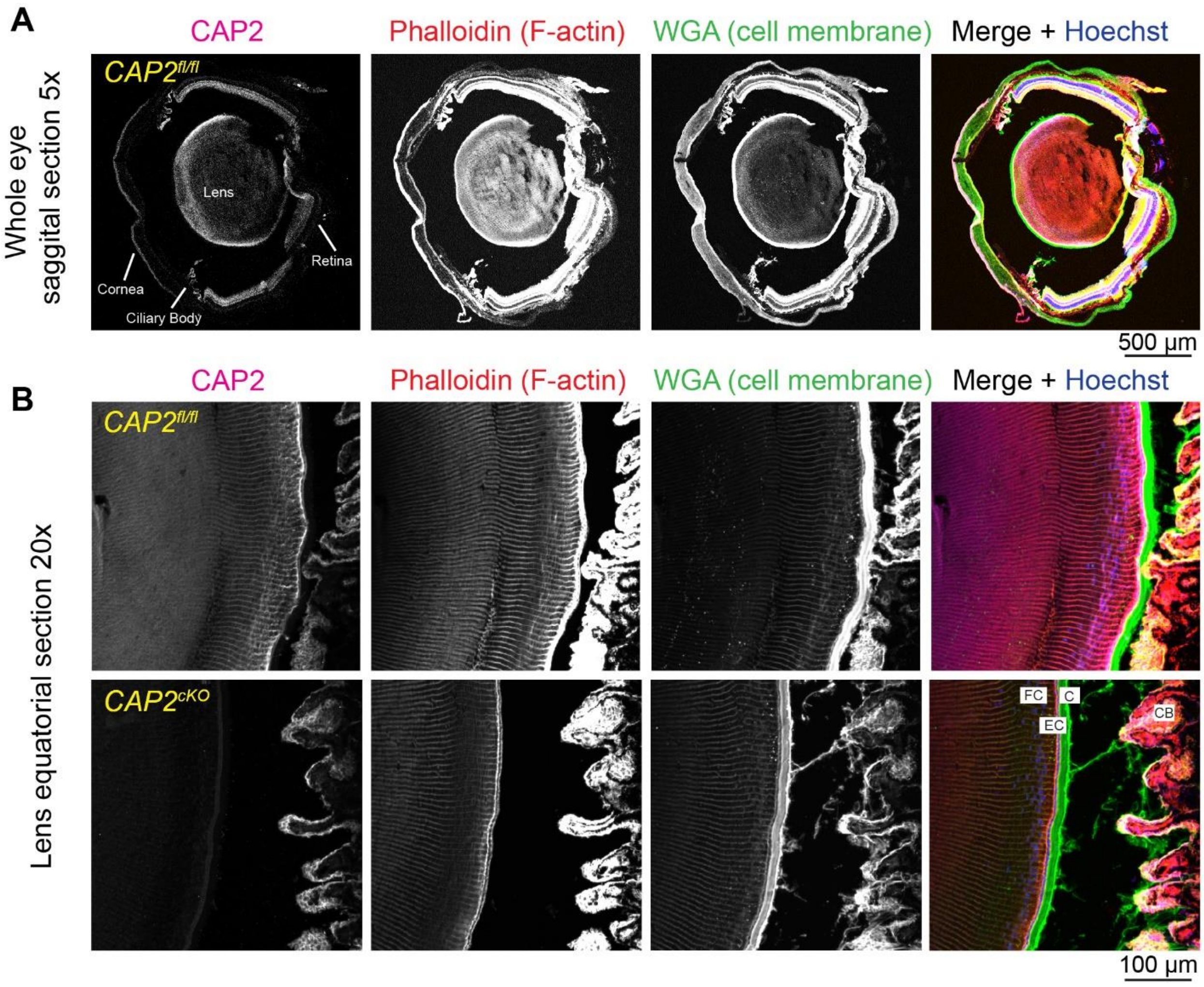

Fig. S3. CAP2 is expressed in multiple ocular tissues but is absent in the lens of *CAP2*^{cKO} mice. (A) Representative 5x confocal images of a sagittal section from an 8-week-old *CAP2*$^{fl/fl}$ and *CAP2*^{cKO} mouse eye immunostained for CAP2 (magenta), F-actin (red), membrane (green), and nuclei (blue). In control eyes (*CAP2*$^{fl/fl}$), CAP2 is detected in the lens, cornea, retina, and ciliary body. (B) Higher-magnification (20×) images of the lens equatorial region show robust CAP2 expression in lens fiber cells and the ciliary body of control mice, but complete loss of CAP2 signal in the lens of *CAP2*^{cKO} mice, confirming tissue-specific deletion. Fiber Cell (FC), Epithelial Cell (EC), Capsule (C), and Ciliary Body (CB). Scale bars: 500 µm (A), 100 µm (B).

Journal of Cell Science • Supplementary information

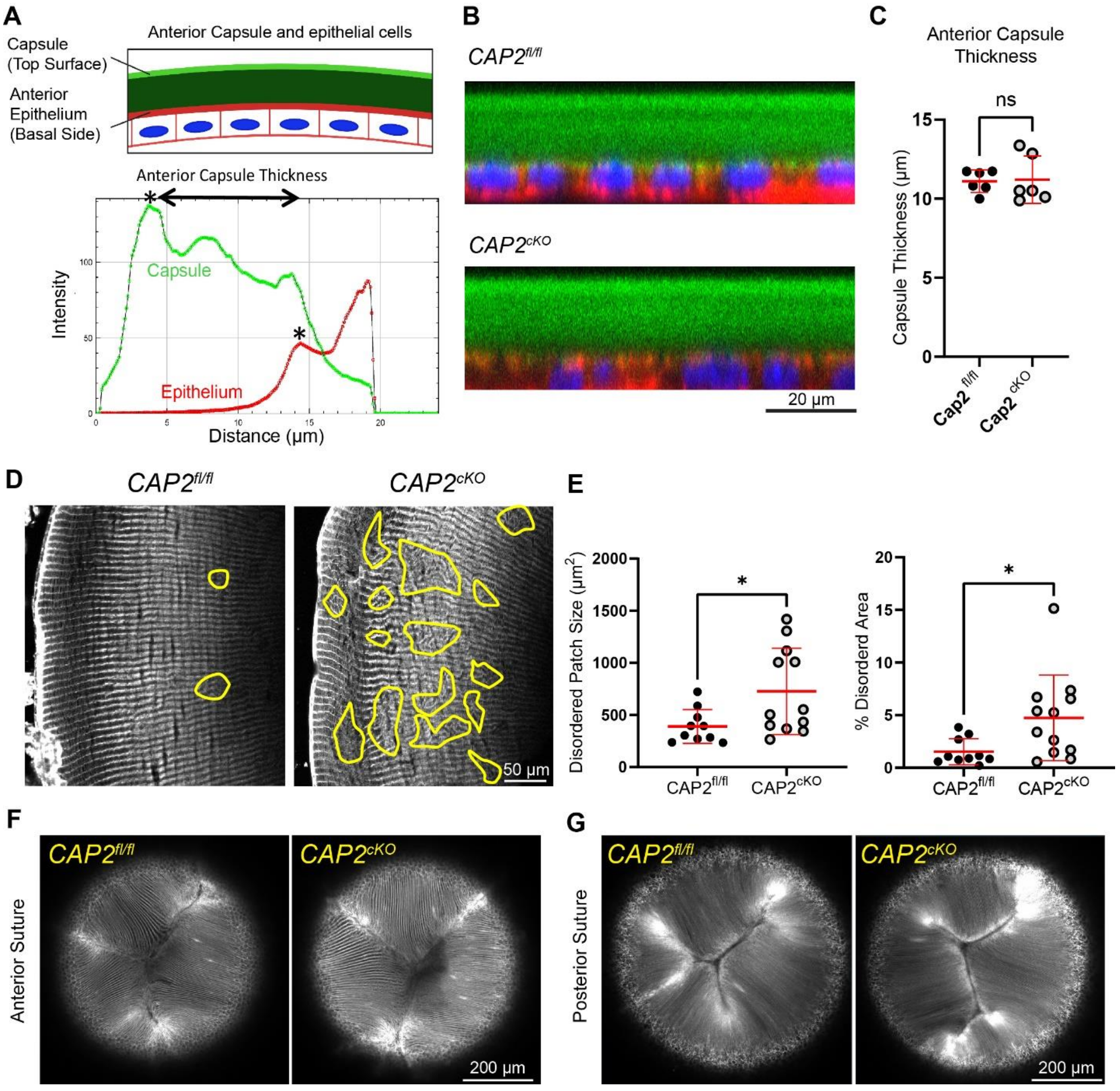

Fig. S4. ***CAP2**^{cKO}* **lenses have normal capsule thickness but mild disorganization of fiber cell packing.** (A) Schematic of the anterior lens capsule and epithelium with a representative intensity profile from a line scan across the capsule (green, WGA) and basal F-actin (red, phalloidin) (B) Confocal X-Z reconstruction of lenses from 6-week-old *CAP2*$^{fl/fl}$ and *CAP2*^{cKO} mice stained with WGA (green), phalloidin(red), and Hoechst (blue). (C) Quantification shows no significant difference in anterior capsule thickness between genotypes (n = 6 lenses from 6 mice per genotype). (D) Equatorial sections of lenses stained with rhodamine-phalloidin reveal disorganized regions of fiber cell packing in *CAP2*^{cKO} lenses, outlined in yellow. (E**)** Quantification of disordered patch size and percent disordered area indicates slightly higher disorganization in *CAP2*^{cKO} lenses than in controls. Plots reflect the mean ± s.d. of 2-3 independent immunostained sections from five different mice per genotype. (F, G) Images of the lens (F) anterior suture and (G) posterior suture. Scale bars: 20 µm (B), 50 µm (D), 200 µm (F, G). *P< 0.05.

Journal of Cell Science • Supplementary information

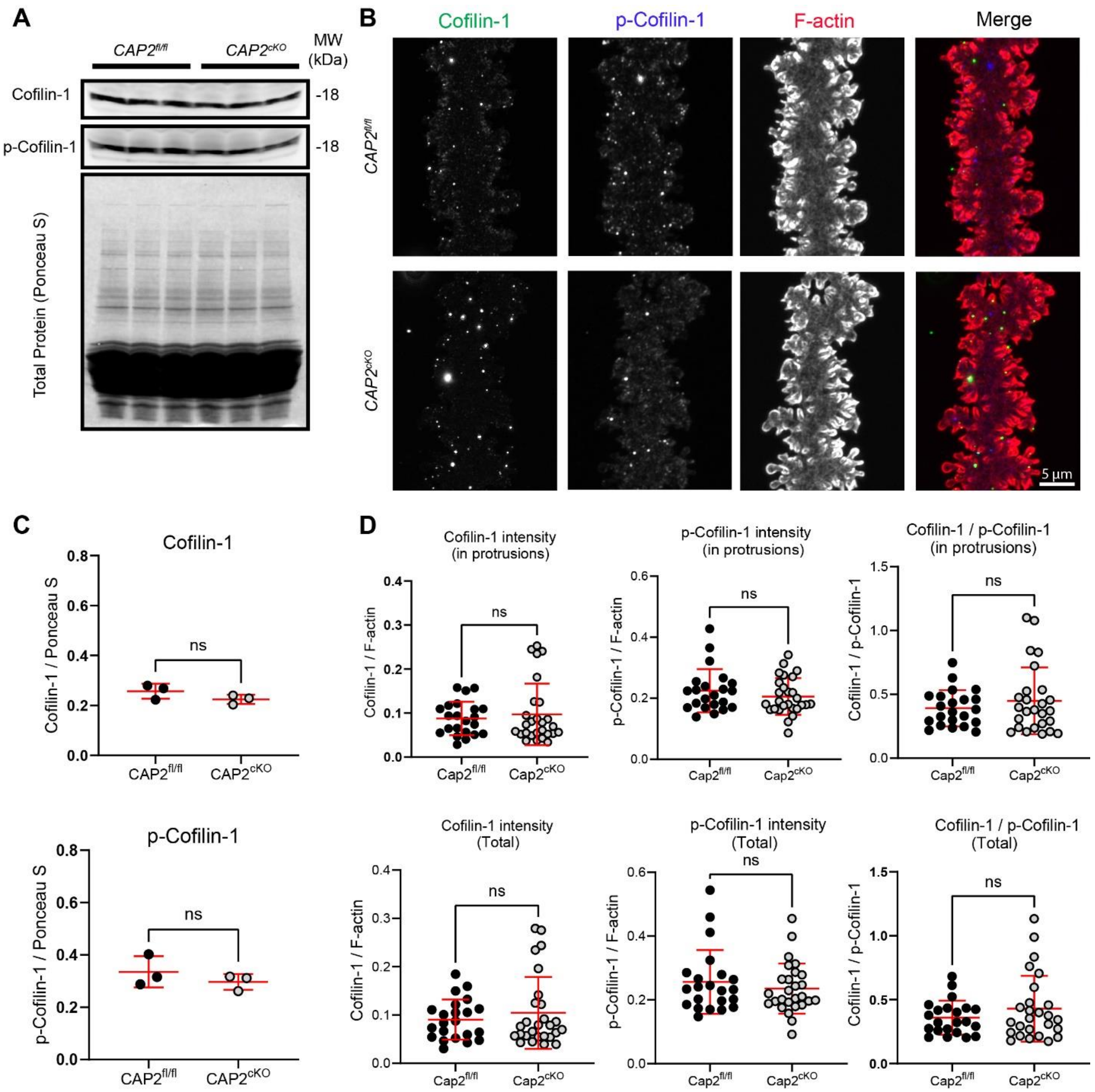

Fig. S5. CAP2 loss does not affect Cofilin activation or localization in the lens. (A) Western blots from whole lenses. (B) Immunostaining of single mature fiber cells from 6-week-old CAP2^{cKO} and CAP2$^{fl/fl}$ lenses for Cofilin-1 (green), p-Cofilin-1 (blue), and F-actin (red). Images are maximum intensity projections. Scale bar: 5 µm. (C) Quantification of proteins from Western blots. Plots show the mean ± s.d. of 3 independent protein samples per genotype. (D) Quantification of puncta intensity with respect to F-actin and the ratio of Cofilin to p-Cofilin puncta intensity. Plots represent the mean ± s.d. of 20-30 individual fibers from 6 lenses of 3 different mice per genotype.

Journal of Cell Science • Supplementary information

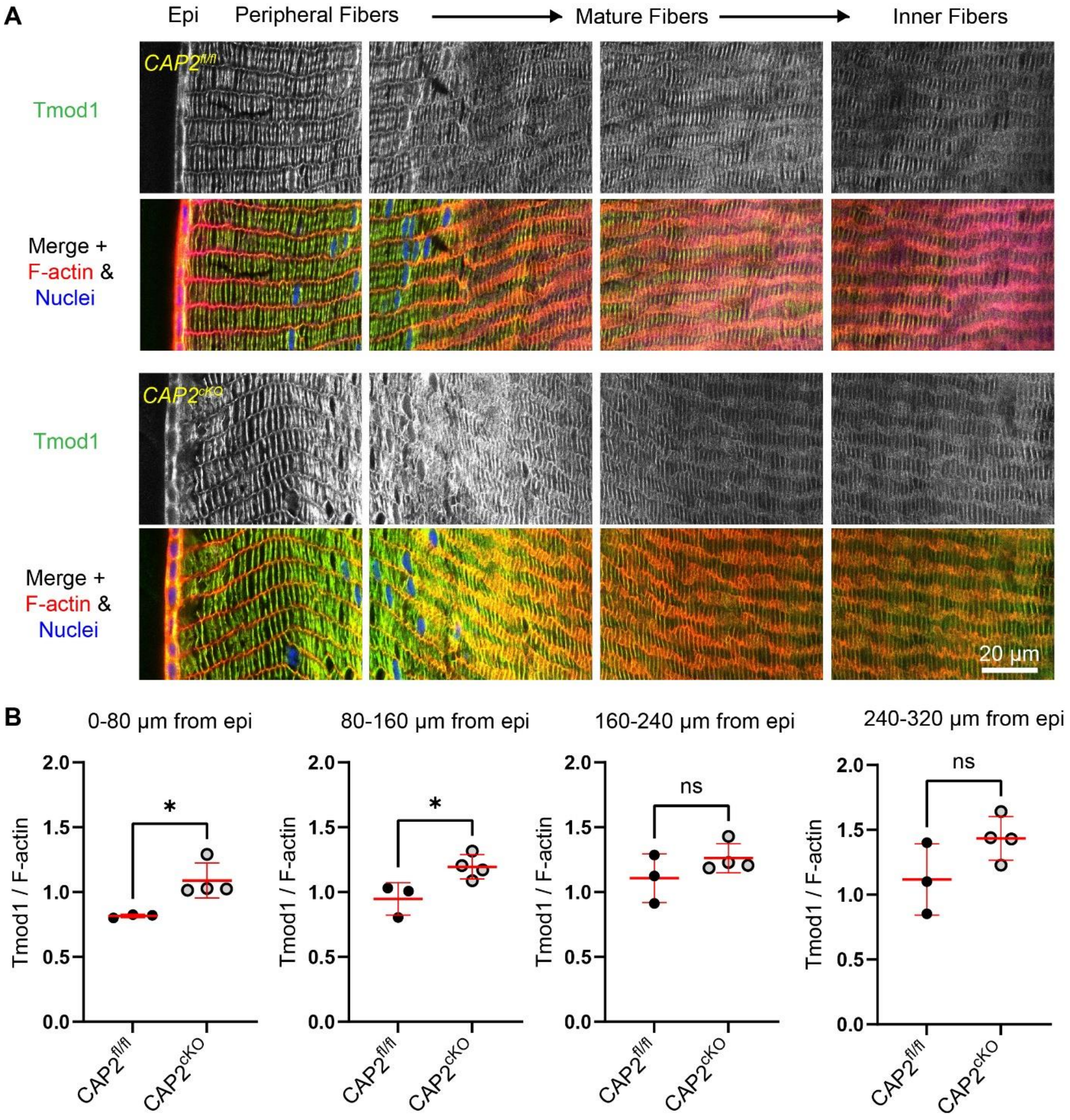

Fig. S6. Increased Tmod1 levels in the outer fiber cell regions of *CAP2^{cKO}* lenses compared to controls. (A) Representative equatorial cryosections from *CAP2$^{fl/fl}$* and *CAP2^{cKO}* mouse lenses stained for Tmod1 (green), F-actin (red), and nuclei (blue). Images are shown across four regions spanning from the epithelial (epi) layer through peripheral, mature, and inner fiber cells. Tmod1 signal appears more intense in the outer fiber cell regions of *CAP2^{cKO}* lenses. Scale bar: 20 µm. (B) Quantification of Tmod1 fluorescence intensity normalized to F-actin in four successive 80-µm regions extending from the lens epithelium. Tmod1 levels were significantly elevated in the 0-80 µm and 80-160 µm regions of *CAP2^{cKO}* lenses compared to controls, while no significant differences were observed in deeper regions (160-240 µm and 240-320 µm). Data are presented as mean ± s.d. from 3-4 biological replicates per group. *P< 0.05.

Journal of Cell Science • Supplementary information

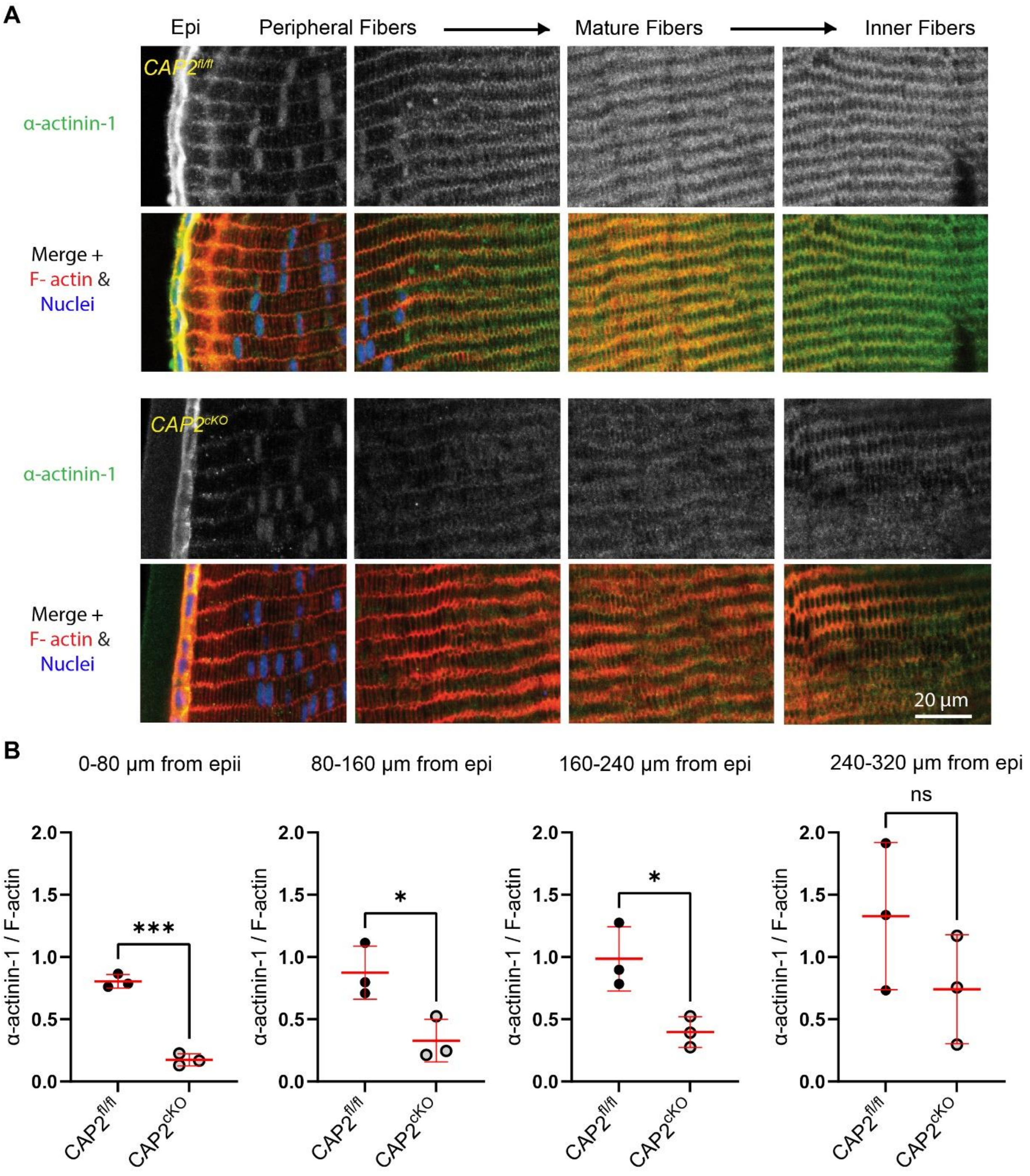

Fig. S7. α-actinin-1 levels are significantly reduced in fiber cells of *CAP2*^{cKO} lenses compared to controls. (A) Representative equatorial cryosections of *CAP2*$^{fl/fl}$ and *CAP2*^{cKO} lenses stained for α-actinin-1 (green), F-actin (red), and nuclei (blue). Sections span from the epithelium through peripheral, mature, and inner fiber cells. α-actinin-1 signal intensity is visibly decreased in CAP2^{cKO} lenses, particularly in the outer and intermediate fiber regions. Scale bar: 20 µm. (B) Quantification of α-actinin-1 fluorescence intensity normalized to F-actin in four successive 80-µm regions from the epithelium (epi). *CAP2*^{cKO} lenses showed significantly reduced α-actinin-1 levels in the 0-80 µm, 80-160 µm, and 160-240 µm regions compared to controls. No significant difference was observed in the deepest region (240-320 µm). Data represent mean ± s.d. from 3 biological replicates per group. *$P < 0.05$; ***$P < 0.001$.

Journal of Cell Science • Supplementary information

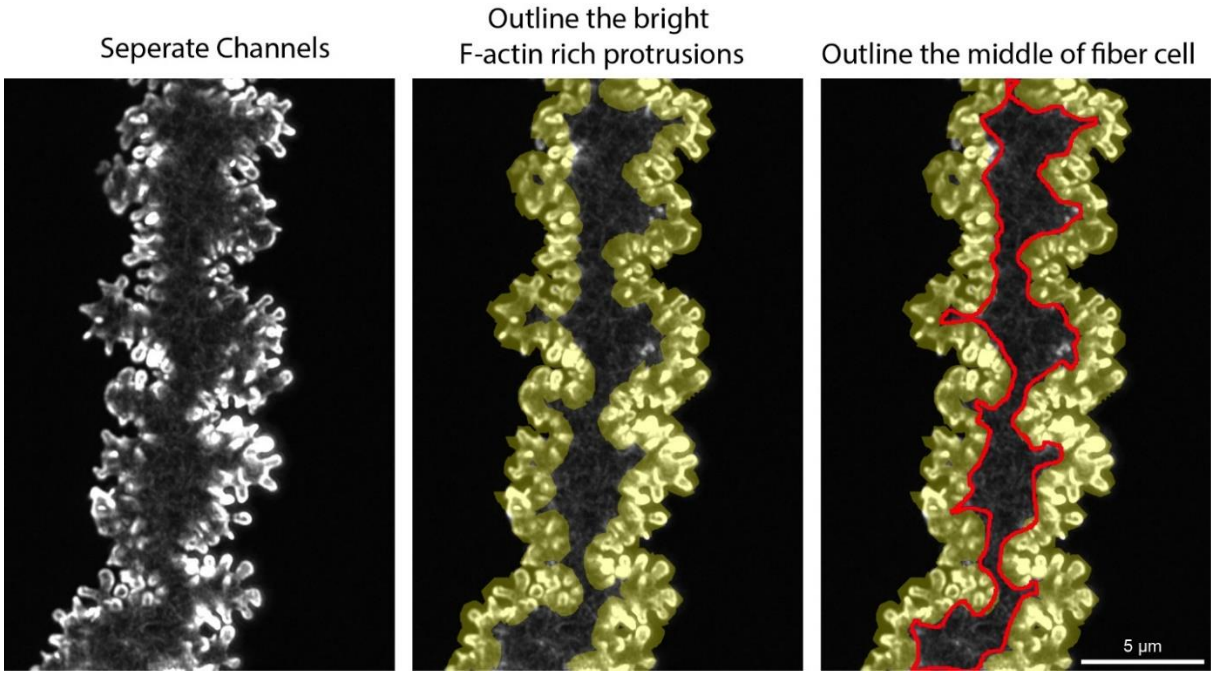

Fig. S8. Method for quantifying fluorescence intensity for the protrusions and cytoplasmic regions of mature lens fiber cells. Representative image of a mature single fiber cell stained with rhodamine phalloidin for F-actin. Left: F-actin channel shown in grayscale. Middle: The bright F-actin-rich protrusion areas were outlined using the freehand line tool in FIJI. Right: The remaining cytoplasmic area was traced using a freehand selection tool (red outline) to identify the central region of the same fiber. Fluorescence intensity was measured separately for the protrusion and the cell body, and the values were summed for total intensity quantification. Scale bar: 5µm.

Journal of Cell Science • Supplementary information

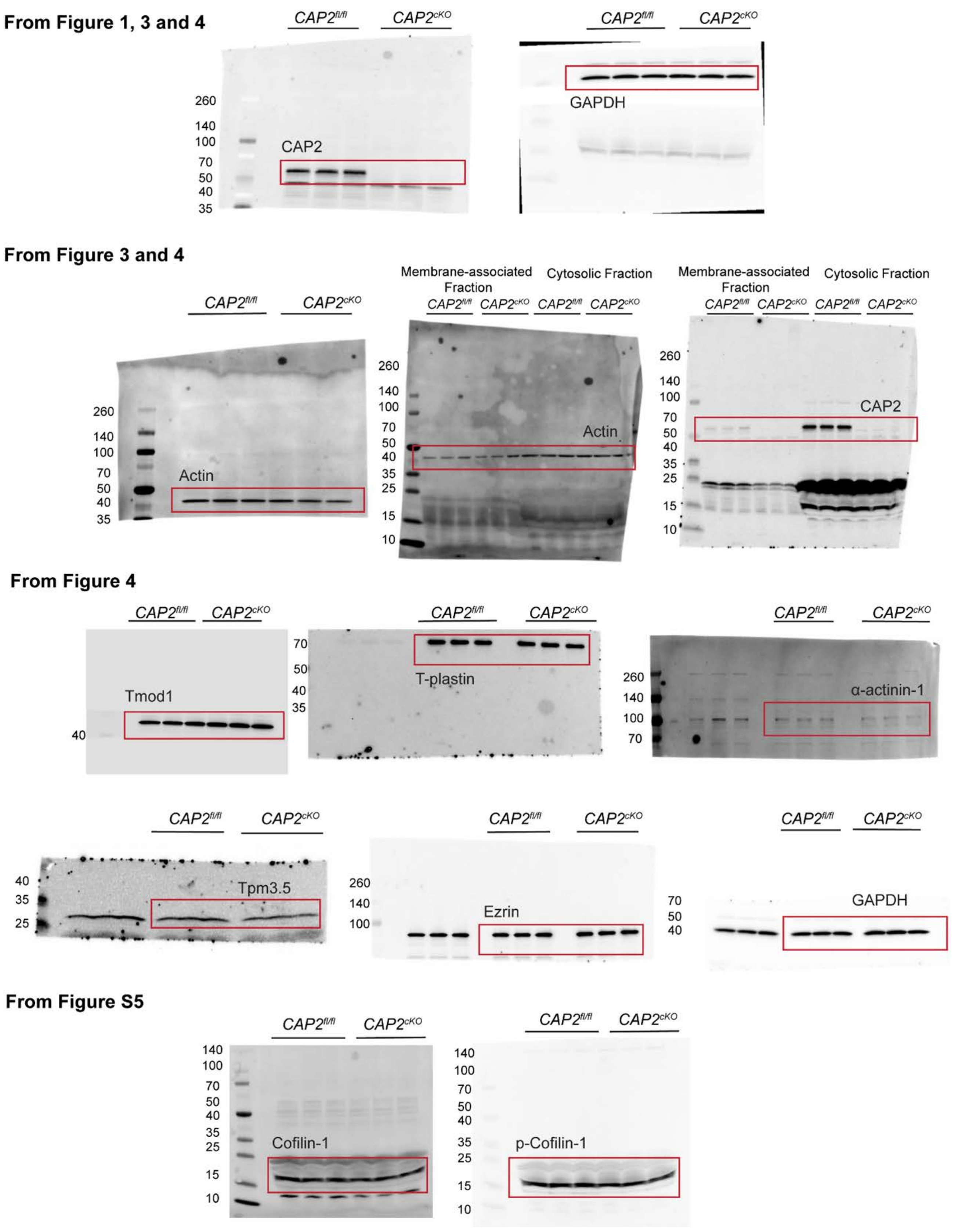

Fig. S9. Blot Transparency

Table S1. List of materials, biochemicals, antibodies, and dyes.

Material	Manufacturer	Catalog #
TRIzol™ Reagent	Thermo Fisher Scientific	15596026
Tris base ((hydroxymethyl)aminomethane)	Thermo Fisher Scientific	BP152-500
HCl (hydrochloric acid)	Sigma-Aldrich	320331
NaCl (sodium chloride)	Thermo Fisher Scientific	S271-500
MgCl2 (magnesium chloride)	Thermo Fisher Scientific	BP214-500
EGTA (ethylene glycol-bis (β-aminoethyl ether)	bioWORLD	40520008-1
NaF (sodium fluoride)	Spectrum	S0167
DTT (Dithiothreitol)	Thermo Fisher Scientific	R0861
Glycine	MP Biomedicals	808822
SDS (sodium dodecyl sulfate)	Sigma-Aldrich	L3771
Methanol	Fisher Scientific	A454-4
Protease Inhibitor Cocktail	Sigma-Aldrich	P8430
Phosphatase Inhibitor	Thermo Fisher Scientific	78420
2X Laemmli sample buffer	BIO-RAD	1610737
Ponceau S solution 0.1% (w/v) in 5% acetic acid	Fluka BioChemica	9189
4–20% linear gradient SDS-PAGE mini-gels	Thermo Fisher Scientific	XP04205BOX
Nitrocellulose membrane	Amersham Protran	10600011
Transfer blot tank	BIO-RAD	1703930
Bovine Serum Albumin (BSA) Fraction V $\geq$98% Purity	Genesee Scientific	25-529
10X phosphate-buffered saline (PBS)	Thermo Fisher Scientific	70011044
Triton™ X-100	Thermo Fisher Scientific	28314
IRDye-680LT-conjugated goat anti-mouse-IgG	LI-COR	926-68020
IRDye-800CW-conjugated goat anti-rabbit-IgG	LI-COR	926-32211
IRDye 680RD Goat anti-Mouse IgM (μ chain specific)	LI-COR	925-68180
Paraformaldehyde (16%)	Electron Microscopy Sciences	15710
Goat Serum	Invitrogen	A11008
Q55 Sonicator Ultrasonic Processor	Qsonica	Mfr # Q55-220
ChemiDoc MP Imaging System	BIO-RAD	12003154
Olympus SZ11 dissecting microscope	Olympus	8F20022
Swiftcam 20 Megapixel Camera	Amazon	SC2003
FluoroDish cell culture dishes	WPI	FD35-100
Sucrose	Sigma-Aldrich	84097-1KG

Journal of Cell Science • Supplementary information

Tissue-Tek OCT Compound	Sakura Finetek	4583
Leica CM3050 cryostat	Leica Biosystems	14903050S03
CrystalCruz® Adhesive Micro Slides	CrystalCruz	sc-363560
Coverslip #1.5 18 x 18 mm (Lens compression assay)	Thermo Fisher Scientific	12-542-AP
Coverslip #11/2 size 22x 40mm	Electron Microscopy Sciences	72204-03
ProLong® Gold antifade reagent	Thermo Fisher Scientific	P36934
Rhodamine phalloidin	Thermo Fisher Scientific	R415
Wheat Germ Agglutinin (WGA) conjugated to Alexa Fluor-488	Thermo Fisher Scientific	W11262
Hoechst 33342	Thermo Fisher Scientific	H3570
Alexa-Fluor-647-conjugated goat anti-rabbit-IgG	Thermo Fisher Scientific	A-21245
Alexa-Fluor-488-conjugated goat anti-mouse-IgG	Thermo Fisher Scientific	A11001
Alexa-Fluor-488-conjugated goat anti-rabbit-IgG	Thermo Fisher Scientific	A11008
Alexa-Fluor-647-conjugated goat anti-mouse-IgG	Thermo Fisher Scientific	A21236
Actin (C4) primary antibody	Millipore	MAB1501
Rabbit Polyclonal Anti-CAP2 antibody	Proteintech	15865-1-AP
Mouse Monoclonal Anti-Tmod1 antibody	Novus Biologicals	NBP2-00955
Mouse monoclonal Anti-Tpm3.5 antibody	DSHB	CH1
Rabbit Polyclonal Anti-T-Plastin antibody	Abcam	ab137585
Mouse monoclonal Anti-α-actinin-1 antibody	Sigma	A5044
Mouse monoclonal Anti-Ezrin antibody	Sigma	E8897
Mouse monoclonal Anti-β2-spectrin antibody	BD Transduction Laboratories	612563
Mouse monoclonal Anti-GAPDH antibody	Novus Biologicals	NB300-221
Mouse monoclonal Anti- Cofilin-1 antibody	Thermo Fisher Scientific	MA5-17275
Rabbit monoclonal Anti - Phospho-Cofilin) (Ser3) antibody	Cell Signaling Technology	3313

Journal of Cell Science • Supplementary information
