# Supplementary material for: Loss of cyclase-associated protein 2 alters actin cytoskeleton organization and biomechanical properties of the ocular lens
Source: J Cell Sci. 2026 May 26;139(10):jcs264491. doi: 10.1242/jcs.264491 (PMC13286373; doi:10.1242/jcs.264491)
Supplement: Supplementary file 1 [file joces-139-264491_review_history.pdf]

Journal of Cell Science

Loss of cyclase-associated protein 2 alters actin cytoskeleton organization and biomechanical properties of the ocular lens

Sepideh Cheheltani, Megan Coffin and Velia M. Fowler
DOI: 10.1242/jcs.264491

Editor: Kathleen Green

Review timeline
Original submission: 6 October 2025
Editorial decision: 5 November 2025
First revision received: 30 March 2026
Accepted: 14 April 2026

Original submission

First decision letter

MS ID#: jcs.264491

MS TITLE: Cyclase associated actin cytoskeleton regulatory protein 2 (CAP2) organizes the actin cytoskeleton to influence the biomechanical properties of the ocular lens

AUTHORS: SEPIDEH CHEHELTANI; Megan Coffin; Velia Fowler
ARTICLE TYPE: Research Article

Dear Dr CHEHELTANI,

We have now reached a decision on the above manuscript.

As you will see, the reviewers raise a number of substantial criticisms that prevent me from accepting the paper at this stage. They suggest, however, that a revised version might prove acceptable, if you can address their concerns. If you think that you can deal satisfactorily with the criticisms on revision, I would be pleased to see a revised manuscript. We would then return it to the reviewers.

Reviewer 1

In the manuscript 'Cyclase associated actin cytoskeleton regulatory protein 2 (CAP2) organizes the actin cytoskeleton to influence the biomechanical properties of the ocular lens', Sepideh Cheheltani and colleagues aims at investigating the function of CAP2 in the ocular lens. The authors generated and analyzed a conditional knockout (cKO) mouse model for CAP2. Analyses included morphology and biomechanics, quantification of actin and actin-binding proteins (ABP) by immunoblots as well as localization/quantification of ABP by immunohistochemistry. The authors found that CAP2 inactivation caused some moderate changes in lens mechanics. These changes were associated with increased levels of the ABP Tmod, Tpm3.5 and T-plastin, while F-actin levels were unchanged and ï¤¡-actinin1 levels were reduced. Based on these findings, the authors suggest a model in which CAP2 inactivation promotes F-actin stabilization and thereby increases lens stiffness and mechanical resilience.

Overall, the manuscript is nicely written and provides interesting novel insights into the molecular mechanisms that control lens biomechanics. In light of the diseases that have recently been

© 2026. Published by The Company of Biologists under the terms of the Creative Commons Attribution License (https://creativecommons.org/licenses/by/4.0/).

associated with human CAP2 mutant variants, it would be highly interesting to know whether the reported defects in cKO causes visual impairments, which unfortunately has not been investigated by the authors.

Apart from this, I only have a few minor concerns:
* CAP2 is abbreviation for 'cyclase-associated protein 2', not for 'cyclase-associated actin cytoskeleton regulatory protein 2'. This term is actually not in common use, and I therefore suggest to delete 'actin cytoskeleton regulatory' in title, abstract, introduction etc
* Apart from promoting actin filament (F-actin) turnover by disassembling ADF/cofilin-decorated filaments, recent studies revealed that CAPs have many more functions with regards to actin regulation. Authors should include these functions in their introduction, and they should take them into account when discussing their own results
* In the introduction, authors refer to CAP2's function in neurons by citing the study by Kumar et al (Front Cell Neurosci, 2016), but unfortunately missed more recent studies (e.g. Pelucchi, Brain Commun 2020; Schuldt, Cell Mol Life Sci 2024)

Reviewer 2

SUMMARY OF THE ADVANCE MADE IN THIS PAPER AND ITS POTENTIAL SIGNIFICANCE TO THE FIELD

This paper addresses the function of the cytoskeleton regulatory protein CAP2 in determining the biomechanical properties of the eye lens. This was achieved by performing a lens specific knockout of the CAP2 gene in the mouse. Changes in lens biomechanics was detected by measuring stiffness under compression and recovery after load removal. Measurements of the levels of actin and binding proteins implicate changes in Tpm3.5, Tmod1 and T-plastin as mediators of the impact of CAP2 knockout. This provides novel insight into the role of CAP2 in regulation of the actin cytoskeleton both in general and in the lens in particular.

SUGGESTIONS TO AUTHORS

Major comments [Please request additional experiments only if they are essential for supporting the conclusions; authors should be encouraged to highlight any claims that are preliminary or speculative, or to discuss any pitfalls or alternative interpretations in a 'Limitations' section]
1. The Abstract concludes that CAP2 regulates cell biomechanics via modulation of actin-binding proteins. There is no evidence presented that CAP2 is a direct regulator of these binding proteins. It is at least equally plausible that knockout of CAP2 leads to changes in filament dynamics which has as its consequence changes in the binding of these proteins to actin filaments. The same concern applies to the title which suggests a directly regulatory role for CAP 2.
2. What are the relative amounts of the two CAP isoforms 1 and 2 in the lens?
3. Figure 1E provides evidence of the juxtaposition of some CAP2 puncta with actin filaments. It would be helpful to perform co-location analysis using Pearson's or Manders' coefficient to quantify this closeness on location.
4. It is not clear how absence of change in nuclear volume or nuclear fraction suggests that CAP2 does not directly regulate lens development or growth. I would expect transcriptomic analysis to be used to support such a suggestion.
5. The data in Figure 3C suggests that there is a decrease in actin in the knockout and that more than 3 samples should be used to perform a statistical test.
6. Both actin and Tpm3.5 appear to be decreased in the knockout samples. Statistical analysis should be provided with more samples or the statement that neither is changed should be more equivocal.
7. The difference in organization of Tmod1 and Tpm3.5 shown in Figure 5 is striking. Do the authors have an explanation given the potential for both to be associated with the same filament? Does this relate to specific subpopulations of filaments?
8. There is no consideration of the impact of the knockout on the location and/or function of nonmuscle myosin 2. Given the biomechanical role of myosin 2, I would expect at least some comment if no data is provided.
9. There is no experimental data that demonstrates that the biomechanical impact of CAP2 is mediated via changes in the binding proteins studies in this paper. It is possible that the key

© 2026. Published by The Company of Biologists under the terms of the Creative Commons Attribution License (https://creativecommons.org/licenses/by/4.0/).

protein change mediating the biomechanical change is none of the studied proteins but rather another protein such as myosin 2. This should be acknowledged by the authors in the discussion.

Minor comments

Reviewer 3

SUMMARY OF THE ADVANCE MADE IN THIS PAPER AND ITS POTENTIAL SIGNIFICANCE TO THE FIELD

The actin cytoskeleton supports the development and function of the eye lens. To date, our knowledge is limited to the role of a handful of actin-stabilizing proteins, whereas the contribution of actin disassembly factors remains elusive. To address this question, this study generated a lens-specific knockout mouse model of Cyclase Associated Protein 2 (CAP2), a protein that is well-known to regulate actin filament disassembly and that leads to microphthalmia in a whole body KO mouse model. They find that loss of CAP2 increases lens stiffness and resilience. Concomitantly, they show that CAP2 KO affects the composition of the actin networks that support lens fiber cells with a specific enrichment in F-actin stabilizing proteins, including Tpm3.5, TMod1, T-Plastin and Ezrin. The CAP2 mechanism proposed is thus comparable to its role in muscle sarcomeres, aka removing TMod1 and Tpms from the filament pointed end such that It becomes available for disassembly and severing by Cofilin. Taken together, this study beautifully shows a novel role for CAP2 in the complex system of the eye lens and extends the many in vitro studies that showed a regulatory role for CAPs at the filament pointed end. However, the intricate relation between CAPs and Cofilin warrant inclusion of information on Cofilins in the absence of CAPs. In addition, the specialized topic of the paper requires extra explanation of the observed effects. Thus, in order to make this paper more accessible for the wide audience of JCS, we request that the authors take the following comments into consideration:

SUGGESTIONS TO AUTHORS

Major comments
1. Given that is it is well established that CAP1/2 are inefficient at disassembling actin filaments by themselves and require Cofilins to do so, the findings of this study would greatly benefit from including information on Cofilin activity for completeness. For this purpose, the authors should assess the amount of active Cofilin (Ser3 phosphorylated versus unphosphorylated Cofilin) by western blot as well as analyze the localization of Cofilin to actin paddles in lens fiber cells by immunofluorescence in wild type and CAP2 KO cells.
2. The difference in lens stiffness, though significant, seems quite small. Given that the eye lens is a very specialized and not widely used model system, the paper would speak to a broader audience if it included an explanation of how this seemingly small change will affect eye sight in the Discussion section.

Minor comments
3. Line 358: T-Plastin can assemble parallel as well as antiparallel bundles/crosslinked networks (see DOI: 10.1073/pnas.2205370119). Please acknowledge this.

First revision

Author response to reviewers' comments

Reviewer 1: In the manuscript 'Cyclase associated actin cytoskeleton regulatory protein 2 (CAP2) organizes the actin cytoskeleton to influence the biomechanical properties of the ocular lens', Sepideh Cheheltani and colleagues aims at investigating the function of CAP2 in the ocular lens. The authors generated and analyzed a conditional knockout (cKO) mouse model for CAP2. Analyses included morphology and biomechanics, quantification of actin and actin-binding proteins (ABP) by immunoblots as well as localization/quantification of ABP by immunohistochemistry. The authors found that CAP2 inactivation caused some moderate changes in lens mechanics. These changes

© 2026. Published by The Company of Biologists under the terms of the Creative Commons Attribution License (https://creativecommons.org/licenses/by/4.0/).

were associated with increased levels of the ABP Tmod, Tpm3.5 and T-plastin, while F-actin levels were unchanged and -actinin1 levels were reduced. Based on these findings, the authors suggest a model in which CAP2 inactivation promotes F-actin stabilization and thereby increases lens stiffness and mechanical resilience.

Overall, the manuscript is nicely written and provides interesting novel insights into the molecular mechanisms that control lens biomechanics. In light of the diseases that have recently been associated with human CAP2 mutant variants, it would be highly interesting to know whether the reported defects in cKO causes visual impairments, which unfortunately has not been investigated by the authors.

Apart from this, I only have a few minor concerns:

* CAP2 is abbreviation for 'cyclase-associated protein 2', not for 'cyclase-associated actin cytoskeleton regulatory protein 2'. This term is actually not in common use, and I therefore suggest to delete 'actin cytoskeleton regulatory' in title, abstract, introduction etc

We corrected all terminology to “Cyclase-associated protein 2 (CAP2)” throughout the title, abstract, main text, and figure legends.

* Apart from promoting actin filament (F-actin) turnover by disassembling ADF/cofilin-decorated filaments, recent studies revealed that CAPs have many more functions with regards to actin regulation. Authors should include these functions in their introduction, and they should take them into account when discussing their own results

We thank the reviewer for bringing this to our attention. We have revised the Introduction and Discussion to include additional CAP functions in actin regulation beyond ADF/cofilin-mediated filament disassembly and to incorporate these roles into our interpretation of the data. (Lines 50-55 and lines 476-478)

* In the introduction, authors refer to CAP2's function in neurons by citing the study by Kumar et al (Front Cell Neurosci, 2016), but unfortunately missed more recent studies (e.g. Pelucchi, Brain Commun 2020; Schuldt, Cell Mol Life Sci 2024)

We have reviewed the recent studies such as Peluchi et al 2020, and have cited them in paragraph of introduction explaining CAP2’s neuronal function in cytoskeletal remodeling in the revised text (Lines 63-66)

Reviewer 2: SUMMARY OF THE ADVANCE MADE IN THIS PAPER AND ITS POTENTIAL SIGNIFICANCE TO THE FIELD

This paper addresses the function of the cytoskeleton regulatory protein CAP2 in determining the biomechanical properties of the eye lens. This was achieved by performing a lens specific knockout of the CAP2 gene in the mouse. Changes in lens biomechanics was detected by measuring stiffness under compression and recovery after load removal. Measurements of the levels of actin and binding proteins implicate changes in Tpm3.5, Tmod1 and T-plastin as mediators of the impact of CAP2 knockout. This provides novel insight into the role of CAP2 in regulation of the actin cytoskeleton both in general and in the lens in particular.

SUGGESTIONS TO AUTHORS

Major comments [Please request additional experiments only if they are essential for supporting the conclusions; authors should be encouraged to highlight any claims that are preliminary or speculative, or to discuss any pitfalls or alternative interpretations in a 'Limitations' section]

1. The Abstract concludes that CAP2 regulates cell biomechanics via modulation of actin-binding proteins. There is no evidence presented that CAP2 is a direct regulator of these binding proteins. It is at least equally plausible that knockout of CAP2 leads to changes in filament dynamics which

© 2026. Published by The Company of Biologists under the terms of the Creative Commons Attribution License (https://creativecommons.org/licenses/by/4.0/).

has as its consequence changes in the binding of these proteins to actin filaments. The same concern applies to the title which suggests a directly regulatory role for CAP 2.

We have revised the abstract and title to state that CAP2 influences actin filament organization and actin-binding protein distribution, avoiding language implying direct regulation. The revised interpretation emphasizes indirect effects via altered filament turnover dynamics.

2. What are the relative amounts of the two CAP isoforms 1 and 2 in the lens?

To address the relative abundance of CAP1 and CAP2 in the lens, we examined publicly available gene expression data from the iSyTE database (https://research.bioinformatics.udel.edu/iSyTE/ppi/). As shown in the screenshots provided in the **response to reviewers**, Cap2 is expressed at consistently higher levels than Cap1 across multiple developmental stages in the lens. When compared to an abundant lens cytoskeletal protein such as ezrin (Ezr), Cap2 expression reaches levels similar to Ezrin during lens development, while Cap1 remains substantially lower throughout lens development.

Select	Symbol	Rank	Dev E10.5 affy430	Dev E11.5 affy430	Dev E12.5 affy430	Dev E16.5 affy430	Dev E17.5 affy430	Dev E19.5 affy430	Dev P0 affy430	Dev P2 affy430	Dev P56 affy430	Dev P28Epi affy430	Dev WEB affy430
	Cap1	-	1398.02	1242.63	1223.24	958.08	792.25	451.85	775.33	945.5	243.5	79.36	1028.8
	Cap2	-	192.49	572.39	1372.33	1317.46	2114.09	1808.86	1779.32	1112.44	1270.78	390.73	104.91
	Ezr	-	1912.92	2345.91	3110.5	2786.01	2419.56	2686.09	2432.16	1652.31	1760.95	1023.43	273.27

In addition, lens-enrichment analysis comparing expression in the lens relative to other tissues (see below) shows that Cap2 is more strongly enriched in the lens than Cap1, particularly during embryonic and early postnatal stages. Together, these data indicate that CAP2 is the predominant CAP isoform expressed in the lens.

Select	Symbol	Rank	Dev E10.5 affy430	Dev E11.5 affy430	Dev E12.5 affy430	Dev E16.5 affy430	Dev E17.5 affy430	Dev E19.5 affy430	Dev P0 affy430	Dev P2 affy430	Dev P56 affy430	Dev P28Epi affy430
	Cap1	-	1.29	1.11	1.2	-1.13	-1.4	-2.64	-1.32	-1.13	-4.5	-12.44
	Cap2	-	1.69	4.49	13.29	12.07	18.83	16.56	16.4	9.97	11.65	4.26
	Ezr	-	6.8	7.95	10.79	9.9	7.9	9.29	8.23	5.75	6.06	3.18

3. Figure 1E provides evidence of the juxtaposition of some CAP2 puncta with actin filaments. It would be helpful to perform co-location analysis using Pearson's or Manders' coefficient to quantify this closeness on location.

We thank the reviewer for this suggestion. To quantitatively assess the spatial proximity between CAP2 puncta and F-actin, we performed co-localization analysis using Manders' coefficient. Using the JaCoP plugin in Fiji, we calculated Manders' coefficient for CAP2 relative to F-actin, which measures the fraction of CAP2 signal overlapping actin-positive pixels. This analysis yielded a value of 0.613 ± 0.139 (mean ± s.d.), indicating that a substantial subset of CAP2 puncta is colocalized with actin filaments. These data have now been added to the Results section. (Lines 161-168)

4. It is not clear how absence of change in nuclear volume or nuclear fraction suggests that CAP2 does not directly regulate lens development or growth. I would expect transcriptomic analysis to be used to support such a suggestion.

In our revised manuscript, we have clarified that our conclusion refers specifically to *gross* lens size and morphology. We now explicitly discuss how lens growth is driven by epithelial-cell proliferation

© 2026. Published by The Company of Biologists under the terms of the Creative Commons Attribution License (https://creativecommons.org/licenses/by/4.0/).

and differentiation (Lines 86-91), and we interpret our morphometric data in this context. (Lines 177-179)

5. The data in Figure 3C suggests that there is a decrease in actin in the knockout and that more than 3 samples should be used to perform a statistical test.

We quantified total actin and Tpm3.5 protein levels from three independent biological replicates per genotype, and statistical analysis revealed no significant differences between control and knockout lenses. Although the representative immunoblots may give the impression of reduced actin levels in the knockout, the quantified data do not support a consistent or statistically significant decrease. The sample size used here is consistent with prior studies analyzing cytoskeletal protein levels in the lens, including the Tpm3.5 study by Cheng et al. (2018, J. Cell Sci. 131: jcs222042), which likewise quantified immunoblot data using three biological replicates. In addition, these findings were reproducible across multiple independent experiments.

6. Both actin and Tpm3.5 appear to be decreased in the knockout samples. Statistical analysis should be provided with more samples or the statement that neither is changed should be more equivocal.

We have revised the figure legend and corresponding text to clarify that, based on quantitative immunoblot analysis of three independent biological replicates per genotype, actin and Tpm3.5 levels were not significantly different between control and knockout lenses. To avoid over-interpretation of the representative blots, we have adjusted the wording to reflect the absence of a statistically significant change rather than asserting an absolute lack of difference.

7. The difference in organization of Tmod1 and Tpm3.5 shown in Figure 5 is striking. Do the authors have an explanation given the potential for both to be associated with the same filament? Does this relate to specific subpopulations of filaments?

We note that the Tmod1 and Tpm3.5 images shown were obtained from different lens fiber cells, rather than from co-labeled single cells, and lens fiber cells exhibit substantial cell-to-cell variability in actin organization in these single fiber cell images. As such, differences in labeling patterns do not necessarily imply association with distinct filament systems.

Previous work has demonstrated that Tmod1 and Tpm3.5 can partially colocalize within punctate structures along F-actin-rich membrane protrusions in mature lens fiber cells (Cheng et al., 2018). Specifically, even in that study, Tmod1 and Tpm3.5 exhibited non-identical distributions, with Tmod1 enriched in valleys between large paddles, whereas Tpm3.5 did not show similar enrichment. Together with the known heterogeneity of fiber cell architecture, these findings support the interpretation that the distinct patterns observed here likely reflect variable actin filament subpopulations, cell-to-cell variability, and technical factors such as antibody accessibility and fixation, rather than mutually exclusive filament associations. We explained this in lines 311-314.

8. There is no consideration of the impact of the knockout on the location and/or function of nonmuscle myosin 2. Given the biomechanical role of myosin 2, I would expect at least some comment if no data is provided.

We agree that nonmuscle myosin II (NMII) is an important regulator of cellular mechanics. Although we did not directly assess NMII localization or function in the CAP2 knockout lenses, prior work by Islam et al. (2023) showed that disruption of NMIIA filament assembly in the lens equatorial epithelium altered epithelial alignment and fiber cell organization without affecting lens size, shape, or stiffness. We have added a statement to the Discussion acknowledging that other cytoskeletal regulators, including NMII, could contribute to lens biomechanics and noting that existing studies suggest perturbation of NMII alone does not necessarily translate into altered whole-lens mechanical properties. (Lines 441-453)

9. There is no experimental data that demonstrates that the biomechanical impact of CAP2 is mediated via changes in the binding proteins studies in this paper. It is possible that the key

© 2026. Published by The Company of Biologists under the terms of the Creative Commons Attribution License (https://creativecommons.org/licenses/by/4.0/).

protein change mediating the biomechanical change is none of the studied proteins but rather another protein such as myosin 2. This should be acknowledged by the authors in the discussion.

We agree that our data do not directly demonstrate the direct cause of biomechanical phenotype is the actin-binding proteins examined, and that other cytoskeletal regulators could contribute. We have revised the Discussion to explicitly acknowledge this possibility and to comment on existing studies examining NM2 function in the lens. (lines 441-453)

Minor comments

Reviewer 3: SUMMARY OF THE ADVANCE MADE IN THIS PAPER AND ITS POTENTIAL SIGNIFICANCE TO THE FIELD

The actin cytoskeleton supports the development and function of the eye lens. To date, our knowledge is limited to the role of a handful of actin-stabilizing proteins, whereas the contribution of actin disassembly factors remains elusive. To address this question, this study generated a lens-specific knockout mouse model of Cyclase Associated Protein 2 (CAP2), a protein that is well-known to regulate actin filament disassembly and that leads to microphthalmia in a whole body KO mouse model. They find that loss of CAP2 increases lens stiffness and resilience. Concomitantly, they show that CAP2 KO affects the composition of the actin networks that support lens fiber cells with a specific enrichment in F-actin stabilizing proteins, including Tpm3.5, TMod1, T-Plastin and Ezrin. The CAP2 mechanism proposed is thus comparable to its role in muscle sarcomeres, aka removing TMod1 and Tpms from the filament pointed end such that It becomes available for disassembly and severing by Cofilin. Taken together, this study beautifully shows a novel role for CAP2 in the complex system of the eye lens and extends the many in vitro studies that showed a regulatory role for CAPs at the filament pointed end. However, the intricate relation between CAPs and Cofilin warrant inclusion of information on Cofilins in the absence of CAPs. In addition, the specialized topic of the paper requires extra explanation of the observed effects. Thus, in order to make this paper more accessible for the wide audience of JCS, we request that the authors take the following comments into consideration:

SUGGESTIONS TO AUTHORS

Major comments

1. Given that is it is well established that CAP1/2 are inefficient at disassembling actin filaments by themselves and require Cofilins to do so, the findings of this study would greatly benefit from including information on Cofilin activity for completeness. For this purpose, the authors should assess the amount of active Cofilin (Ser3 phosphorylated versus unphosphorylated Cofilin) by western blot as well as analyze the localization of Cofilin to actin paddles in lens fiber cells by immunofluorescence in wild type and CAP2 KO cells.

Thank you for this excellent suggestion. To address this point, we assessed cofilin activity and localization in control and CAP2 knockout lenses. Western blot analysis showed no significant differences in total cofilin or Ser3-phosphorylated (inactive) cofilin levels between genotypes (Fig. S5A, C). In addition, immunofluorescence analysis of isolated lens fiber cells revealed comparable localization of cofilin and phospho-cofilin relative to actin-rich membrane protrusions in control and CAP2 knockout lenses (Fig. S5B, D). These results have been added to the Results section (Lines 247-262).

2. The difference in lens stiffness, though significant, seems quite small. Given that the eye lens is a very specialized and not widely used model system, the paper would speak to a broader audience if it included an explanation of how this seemingly small change will affect eye sight in the Discussion section.

Although mice lack accommodative ability, lens stiffness remains a critical determinant of lens shape, internal strain distribution, and refractive index organization. Thus, even in a non-accommodating system, changes in lens mechanical properties can influence optical quality and provide insight into fundamental structure and function relevant to vision (Cheng et al 2019, Aging,

© 2026. Published by The Company of Biologists under the terms of the Creative Commons Attribution License (https://creativecommons.org/licenses/by/4.0/).

doi: 10.18632/aging.102584). We have added a paragraph to the Discussion to clarify this point. (Lines 377-386)

Minor comments

3. Line 358: T-Plastin can assemble parallel as well as antiparallel bundles/crosslinked networks (see DOI: 10.1073/pnas.2205370119). Please acknowledge this.

We thank the reviewer for this clarification. We have revised the text to acknowledge that T-plastin can assemble both parallel and antiparallel actin bundles and cross-linked networks.

Second decision letter

MS ID#: jcs.264491R1

MS Title: Loss of cyclase-associated protein 2 (CAP2) alters actin cytoskeleton organization and biomechanical properties of the ocular lens

Authors: SEPIDEH CHEHELTANI; Megan Coffin; Velia Fowler
Article Type: Research Article

Dear Dr CHEHELTANI,

I am happy to tell you that your manuscript has been accepted for publication in Journal of Cell Science, pending standard publication integrity checks.

© 2026. Published by The Company of Biologists under the terms of the Creative Commons Attribution License (https://creativecommons.org/licenses/by/4.0/).
